# RPamide neuropeptides NLP-22 and NLP-2 act through GnRH-like receptors to promote sleep and wakefulness in *C. elegans*

**DOI:** 10.1038/s41598-020-66536-2

**Published:** 2020-06-18

**Authors:** Petrus Van der Auwera, Lotte Frooninckx, Kristen Buscemi, Ryan T. Vance, Jan Watteyne, Olivier Mirabeau, Liesbet Temmerman, Wouter De Haes, Luca Fancsalszky, Alexander Gottschalk, David M. Raizen, Matthew D. Nelson, Liliane Schoofs, Isabel Beets

**Affiliations:** 1 Department of Biology, KU Leuven, Naamsestraat 59, 3000 Leuven, Belgium; 2 Department of Biology, Saint Joseph’s University, 5600 City Ave, Philadelphia, PA 19131 USA; 3 Institut Curie, Inserm U830, 26 rue d’Ulm, 75248 Paris, France; 4 Buchmann Institute for Molecular Life Sciences (BMLS), Goethe University, Max-von-Laue-Strasse 15, D-60438 Frankfurt, Germany; 5 Department of Neurology, Perelman School of Medicine, University of Pennsylvania, 415 Curie Blvd, Philadelphia, PA 19104 USA

**Keywords:** Neurophysiology, Peptides

## Abstract

Sleep and wakefulness are fundamental behavioral states of which the underlying molecular principles are becoming slowly elucidated. Transitions between these states require the coordination of multiple neurochemical and modulatory systems. In *Caenorhabditis elegans* sleep occurs during a larval transition stage called lethargus and is induced by somnogenic neuropeptides. Here, we identify two opposing neuropeptide/receptor signaling pathways: NLP-22 promotes behavioral quiescence, whereas NLP-2 promotes movement during lethargus, by signaling through gonadotropin-releasing hormone (GnRH) related receptors. Both NLP-2 and NLP-22 belong to the RPamide neuropeptide family and share sequence similarities with neuropeptides of the bilaterian GnRH, adipokinetic hormone (AKH) and corazonin family. RPamide neuropeptides dose-dependently activate the GnRH/AKH-like receptors GNRR-3 and GNRR-6 in a cellular receptor activation assay. In addition, *nlp-22*-induced locomotion quiescence requires the receptor *gnrr-6*. By contrast, wakefulness induced by *nlp-2* overexpression is diminished by deletion of either *gnrr-3* or *gnrr-6*. *nlp-2* is expressed in a pair of olfactory AWA neurons and cycles with larval periodicity, as reported for *nlp-22*, which is expressed in RIA. Our data suggest that the somnogenic NLP-22 neuropeptide signals through GNRR-6, and that both GNRR-3 and GNRR-6 are required for the wake-promoting action of NLP-2 neuropeptides.

## Introduction

Sleep is an essential quiescent state, conserved at the molecular level across distantly related animals^1–5^. Because animals display a remarkable diversity of sleep traits, a consensus definition for sleep-like states has been set based on behavioral changes shared with human sleep. These include behavioral quiescence, reduced sensory responsiveness, reversibility, the assumption of a specific posture, and homeostatic regulation^1,4,6,7^. Sleep deprivation is detrimental to diverse biological processes, including metabolism, longevity, and memory formation^8–11^.

Genetic studies in model organisms such as mice, zebrafish*, Drosophila* and *C. elegans* have provided powerful ways to dissect core mechanisms of sleep-like states that are evolutionarily conserved across these species^1–3,6,10^. A well-known example is the circadian protein PERIOD that regulates the timing of sleep^12,13^. Other conserved sleep pathways include epidermal growth factor (EGF) and notch signaling^14–16^. Conserved wake-promoting pathways include dopamine and pigment dispersing factor (PDF) signaling^17–19^. How these sleep and wake pathways interact is still unclear (for review, see^3,4,18^). Mounting evidence indicates that sleep-wake transitions require the coordination of several brain regions and engage multiple neurochemical systems, including biogenic amines^1,17,20^ and neuropeptides^19,21^. In mammals, hypothalamic orexin/hypocretin neuropeptides promote wakefulness, while galanin neuropeptides and melanin-concentrating hormone (MCH) are involved in REM sleep^22,23^. In zebrafish, the neuropeptides neuromedin U and neuropeptide Y are wake- and sleep-promoting, respectively^24,25^. In *Drosophila*, the neuropeptides amnesiac, myoinhibitory peptide, neuropeptide F, short neuropeptide F and SIFamide all promote sleep^19,26,27^, whereas PDF promotes arousal^28–30^.

The nematode *Caenorhabditis elegans* sleeps during lethargus, a period of behavioral quiescence that occurs before each larval molt and that meets behavioral criteria of sleep^2,31–37^. *C. elegans* lethargus has been characterized as a global quiescent brain state with distinct gene expression in sleep-active neurons^37–39^. Many of the sleep-regulatory pathways identified in vertebrates and insects are conserved in *C. elegans* and sleep-like quiescence during lethargus shows fundamental similarities to sleep in other animals^4^. Neuropeptidergic signaling systems conserved in *C. elegans* comprise the PDF orthologous system PDF-1/PDFR-1 and the RFamide neuropeptide system FLP-2/FRPR-18, which promote arousal by increasing sensory activity^30,40^. Inhibition of these wake-promoting neuropeptides by FLP-18/NPR-1 and FLP-21/NPR-1 signaling reduces sensory responsiveness during lethargus^21^. Two other neuropeptides are known to play a somnogenic role in lethargus: FLP-11, expressed in the GABAergic RIS interneuron, and NLP-22, expressed in the glutamatergic RIA interneurons^39,41^. FLP-11 seems to signal through multiple receptors including FRPR-3, NPR-4 and NPR-22^39^, whereas the molecular target(s) of NLP-22 have remained elusive.

The established role of RFamide neuropeptides as regulators of sleep in both *C. elegans* and *Drosophila* led to the discovery of a sleep-promoting function for the hypothalamic RFamide neuropeptide VF (NPVF also known as RFRP-1/2/3) in zebrafish larvae^42^. NPVF is also called Gonadotropin-Inhibitory Hormone (GnIH) because it suppresses Gonadotropin-Releasing Hormone (GnRH) synthesis and release^43^. Accumulating evidence indicates that also GnRH-like signaling regulates sleep in the central nervous system. In *Drosophila*, GnRH-like signaling is required for starvation-induced sleep suppression^44,45^. In addition, a likely downstream effector of this GnRH-like signaling pathway, salt-inducible kinase 3 (SIK3), is a conserved regulator of sleep^46–48^. Strong interconnections between GnRH signaling and the hypocretin/orexin neuronal circuits controlling sleep/wake states have been reported in vertebrates^49,50^. Human patients with primary insomnia also display altered GnRH levels^51^.

In 2009, we discovered that an adipokinetic hormone (AKH)-like neuropeptide signals through a GnRH-like receptor in *C. elegans*^52^. Based on this finding, we postulated that the insect AKH and the vertebrate GnRH systems share a common evolutionary origin in bilaterian animals^52,53^. Additional studies later confirmed that AKH, corazonin and GnRH indeed belong to the same superfamily of GnRH-like neuropeptides, members of which occur in all bilaterian animals^54–56^. GnRH/AKH-like peptides are involved in energy homeostasis^57^ and control carbohydrate and lipid metabolism in insects^58^. In *C. elegans* a recent study showed that lipid mobilization promotes sleep^59^. These data, together with the growing evidence for a role of vertebrate GnRH in the regulation of sleep, led us to hypothesize that *C. elegans* GnRH-like signaling may be involved in sleep regulation. The *C. elegans* genome encodes eight GnRH-like G protein-coupled receptors (GPCRs)^60,61^, the majority of which is still orphan, i.e. an endogenous ligand has not yet been identified. Here we show that two of these GNRRs are activated by the RPamide neuropeptides NLP-22 and NLP-2, displaying sequence similarities to GnRH/AKH-like peptides, and demonstrate that they act opposingly to control sleep and wakefulness in *C. elegans*.

## Results

### The *C. elegans* genome encodes eight GnRH/AKH-related receptors

**Figure 1:**
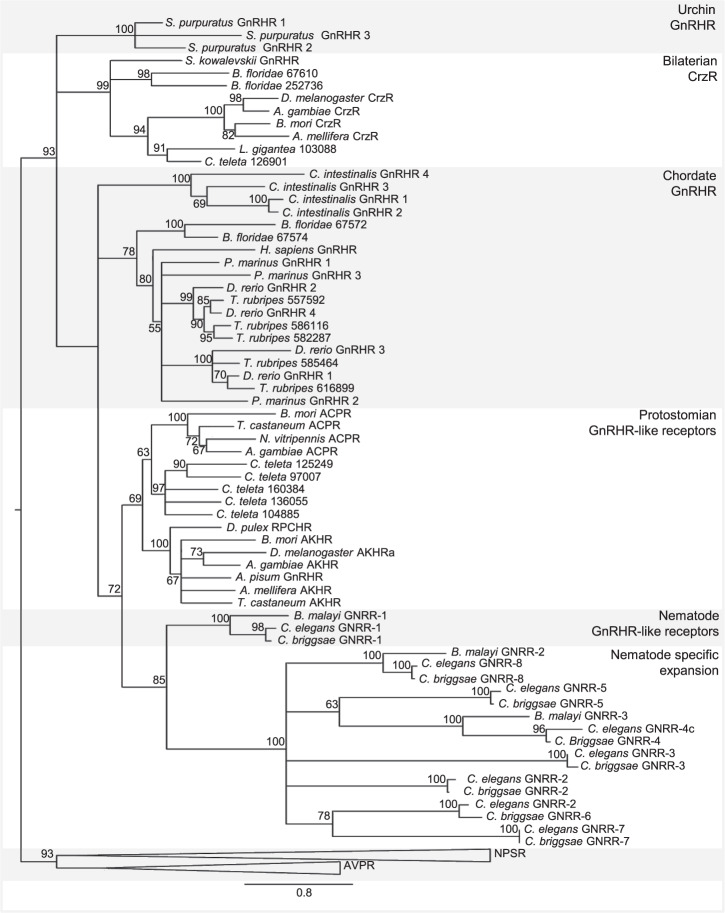
Maximum likelihood tree of vertebrate and invertebrate GnRH/AKH receptors. Branch lengths indicate the expected number of substitutions per site. Node numbers are branch support values (%) derived from 100 non-parametric bootstraps. Accession numbers are provided in Materials and Methods. ACPR, adipokinetic hormone/corazonin related peptide receptor; AKHR, adipokinetic hormone receptor; AVPR, arginine vasopressin receptor; CrzR, corazonin receptor; GnRHR, gonadotropin-releasing hormone receptor; GNRR, gonadotropin-releasing hormone receptor related receptor; RPCHR, red-pigment concentrating hormone receptor; NPSR, neuropeptide S receptor.

Using characterized GnRH/AKH receptors as a query in a protein BLAST search^62^, we identified eight putative GnRH/AKH-like receptors in *C. elegans* (GNRR-1 to GNRR-7 and DAF-38/GNRR-8). Phylogenetic analysis showed that these receptors are orthologs of the GnRH/AKH receptor family, as they cluster together with other ecdysozoan GnRH/AKH receptors (Fig. 1). The nematode cluster can be subdivided in two groups consisting of GNRR-1, which is located more basal to the clade node, and a paralogous group formed by 7 other GnRH/AKH-like receptors. GNRR-1 was identified as a receptor for NLP-47, a GnRH/AKH neuropeptide ortholog in *C. elegans*, and has been the only characterized GNRR so far^52^. The other GnRH/AKH-like receptors are still orphan receptors, i.e. GPCRs with no known peptide ligand. Only DAF-38/GNRR-8 is known to mediate the response to ascaroside pheromones that control dauer entry when it heterodimerizes with the DAF-37 chemoreceptor^63^.

### RPamide neuropeptides activate GNRR-3 and GNRR-6 *in vitro*

**Figure 2:**
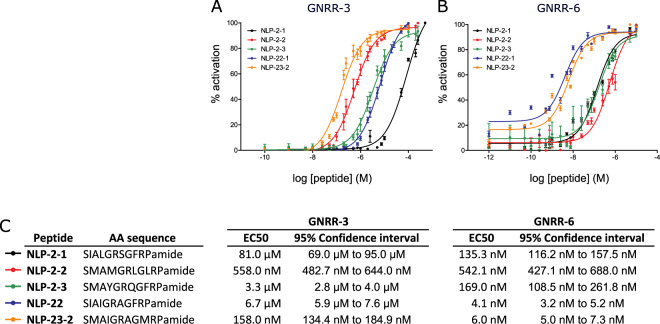
NLP-2, NLP-22, and NLP-23 peptides activate GNRR-3 and GNRR-6 *in vitro*. Dose-response curves for GNRR-3 (**A**) and GNRR-6 (**B**) co-expressed in CHO cells with a promiscuous Gα_16_ protein are shown as relative (%) to the highest value (100% activation) after normalization to the total calcium response. Each data point represents the mean ± SEM of N = 5–7 replicates for each peptide. (**C**) Amino acid (AA) sequences of RPamide neuropeptides activating GNRR-3 and GNRR-6 with their respective mean EC_50_ values and 95% Confidence intervals.

Transmembrane topology prediction revealed that GNRR-1 to −3, GNRR-5 to −7 and DAF-38/GNRR-8 have seven alpha-helical transmembrane domains, typical for GPCRs (Supplementary Fig. S1). We tested a *C. elegans* peptide library for the ability to activate these seven receptors using an *in vitro* calcium mobilization assay. We cloned and transiently expressed each of the seven GNRRs in Chinese hamster ovary (CHO) cells stably expressing apo-aequorin and the human promiscuous Gα_16_ protein. These cells were challenged with a synthetic library of over 340 *C. elegans* peptides of the RFamide (FLP) and neuropeptide-like protein (NLP) families^64^. Besides the known NLP-47/GNRR-1 interaction^52^, only GNRR-3 and GNRR-6 displayed a functional response in this assay (Fig. 2). Of all peptides tested, only peptides encoded by the genes *nlp-2* (NLP-2-1, NLP-2-2, NLP-2–3), *nlp-22* (NLP-22) and *nlp-23* (NLP-23-2) activated these receptors in a dose-dependent manner, although with different potencies. Peptides encoded by *nlp-2* and *nlp-23* potently activated GNRR-3 with EC_50_ values in the nanomolar range. By contrast, NLP-22 activated GNRR-3 with far lower potency (EC_50_ value > 6 µM), which may be physiologically irrelevant. GNRR-6 was potently activated by NLP-22 and NLP-23 peptides. Peptides encoded by *nlp-2* also activated GNRR-6, although with a higher EC_50_ value than NLP-22 and NLP-23. None of these neuropeptides elicited a calcium response in cells transfected with an empty vector as a negative control, indicating that the responses are specific to the expressed receptors (Supplementary Fig. S2). When GNRR-3 or GNRR-6 were expressed in cells devoid of the promiscuous Gα_16_ subunit, a dose-dependent increase in aequorin luminescence was still observed upon addition of their peptide ligands, suggesting that GNRR-3 and GNRR-6 can couple to Gα_q_ proteins expressed in these cells to elicit a calcium response (Supplementary Fig. S3). In short, we identified the neuropeptides encoded by *nlp-2*, *nlp-22* and *nlp-23* as bioactive ligands of the GnRH/AKH-like receptors GNRR-3 and GNRR-6 *in vitro*.

### RPamide neuropeptides display sequence similarities to GnRH/AKH

**Figure 3:**
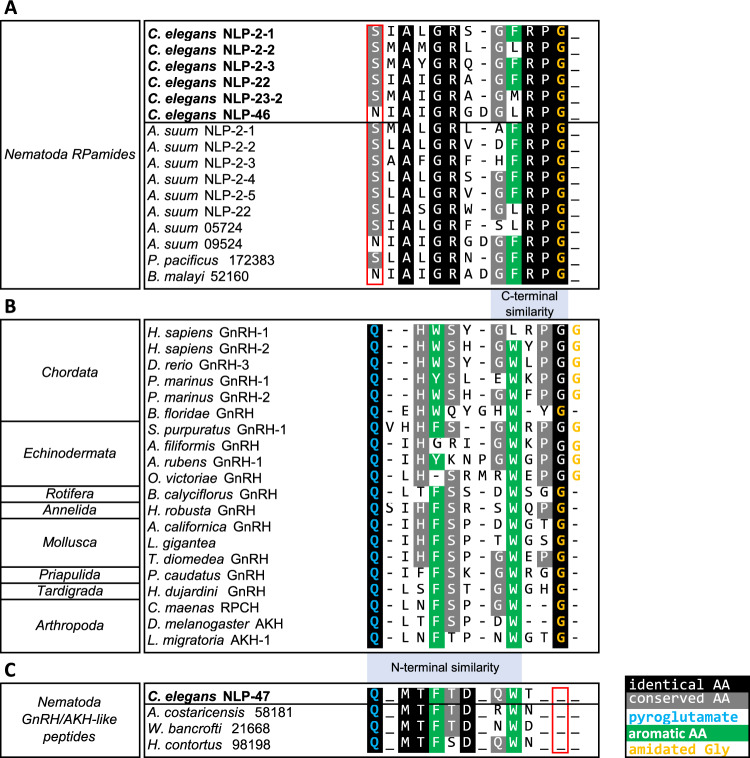
RPamide peptides are conserved among nematodes and share sequence similarity with GnRH/AKH peptides. (**A**) Amino acid sequence alignment of nematode RPamide neuropeptides. All have a C-terminal glycine amidation target but lack an N-terminal pyroglutamate. (**B**) Amino acid sequence alignment of GnRH/AKH peptides across major animal phyla. (**C**) Amino acid sequence alignment of nematode GnRH/AKH-like NLP-47 peptides lacking the C-terminal glycine amidation target. For **A–C**, residues with a colored background are conserved in at least 50% of the sequences. Identical residues are depicted in black, conserved residues in grey and conserved aromatic residues in green. Pyroglutamate residues are indicated in blue and amidated glycine residues are indicated in orange. Absence of these modifications in nematode RPamide or GnRH peptides, respectively, are indicated by red boxes. Hyphens indicate gaps and a more elaborate alignment is depicted in Supplementary Fig. S5.

*C. elegans* neuropeptides encoded by *nlp-2*, *nlp-22* and *nlp-23* share a C-terminal FRPG motif, in which the C-terminal glycine provides a target for amidation *in vivo*, hereby generating FRPamide neuropeptides. The three genes are clustered on the X chromosome (Supplementary Fig. S4), suggesting that they arose from tandem gene duplications. Pattern and BLAST analyses of the FRPamides highlighted that also NLP-46 is a possible member of this RPamide neuropeptide family, which is evolutionarily well conserved among nematodes (Fig. 3A) and characterized by a C-terminal RPamide motif. The predicted neuropeptides encoded by *nlp-2*, *nlp-22*, *nlp-23* and *nlp-46* have recently been identified by mass spectrometry analysis^65^, indicating that the predictions are correct. Besides the conserved C-terminus, nematode RPamides typically have an alanine residue at position three and conserved glycine and arginine residues at positions five and six, respectively (Fig. 3A).

Many neuropeptidergic signaling systems are conserved throughout the Animal Kingdom and several orthologous neuropeptide-receptor pairs have been identified^64,66–68^. In an attempt to deduce the phylogenetic origin of the nematode RPamides, we looked for degenerate motifs shared between RPamides and members of other known neuropeptide families. This search revealed a motif (G[F/W]XPG) near the C-terminus that is found in several members of the urbilaterian conserved GnRH/AKH neuropeptide family (Fig. 3B and Supplementary Fig. S5). Nematode GnRH/AKH-like neuropeptides derived from NLP-47, which activate the GnRH/AKH receptor ortholog GNRR-1^52^, lack this characteristic C-terminal motif. By contrast, NLP-47 peptides share an N-terminal pyroglutamate residue and [FW]-[ST]-X2-W motif with the GnRH/AKH peptide family that is absent in RPamides (Fig. 3C). One of the NLP-23 derived peptides (NLP-23-1, LYISRQGFRPA) also lacks the C-terminal glycine residue of RPamides. In contrast to amidated NLP-2, NLP-22 and NLP-23 derived neuropeptides, NLP-23-1 and GnRH-like neuropeptides derived from NLP-46 and NLP-47 did not activate GNRR-3 or GNRR-6 *in vitro* (data not shown).

### NLP-22 RPamide neuropeptides induce locomotion quiescence through GNRR-6

**Figure 4:**
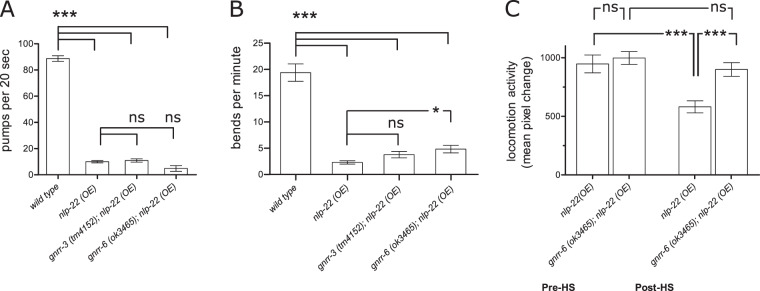
GNRR-6, but not GNRR-3, is required *for nlp-22* induced locomotion quiescence. (**A**,**B**) Heat-shock induced overexpression of *nlp-22* reduces both pharyngeal pumping (**A**) (N > 10 animals) and body bending (**B**) (N > 18 animals) compared to wild-type animals. These *nlp-22* induced quiescence phenotypes are unaffected in *gnrr-3* mutant animals. Overexpression of *nlp-22* in *gnrr-6* mutants attenuates locomotory activity, showing significantly more body bends, while *nlp-22* induced feeding quiescence is still adequate in *gnrr-6* mutants. (**C**) Long-term behavioral tracking before and after heat shock (HS) induction of *nlp-22* overexpression shows that *gnrr-6* mutants display deficient movement quiescence compared to *nlp-22* overexpression in wild-type animals (N = 24 animals). Error bars indicate SEM. One-way ANOVA and Tukey test; ***P < 0.001; *P < 0.05; ns, not significant (*P* > 0.05).

The RPamide neuropeptide NLP-22 promotes movement and feeding quiescence in *C. elegans*^41^. Since GNRR-3 and GNRR-6 are activated by RPamide neuropeptides *in vitro*, we asked whether these GPCRs are involved in sleep regulation. If NLP-22 transduces its behavioral effects through GNRR-3 and/or GNRR-6, loss-of-function of *gnrr-3* and/or *gnrr-6* should abrogate the somnogenic effects of *nlp-22*. To test this, we quantified the effect of *gnrr-3* and *gnrr-6* mutations (Supplementary Fig. S4a) on behavioral quiescence of adult worms overexpressing *nlp-22* from a heat-shock inducible promoter^41^, by counting the number of body bends and pharyngeal pumps. Overexpression of *nlp-22* in *gnrr-3* mutant adults reduced pharyngeal pumping and body bending activity to the same degree as observed in a wild-type background (Fig. 4A,B), suggesting that GNRR-3 is not an endogenous receptor for NLP-22 in the regulation of behavioral quiescence. Similarly, the suppression of pharyngeal pumping induced by *nlp-22* was not affected in mutants of *gnrr-6* (Fig. 4A). By contrast, adult *gnrr-6* mutants overexpressing *nlp-22* had a small but significant elevation of body bend frequency in comparison to animals overexpressing *nlp-22* in a wild-type background (Fig. 4B). We further examined the potential effect of *gnrr-6* on *nlp-22*–induced locomotion quiescence by quantifying movement before and after heat shock-induced expression of *nlp-22* using the WorMotel, an automated machine vision-based platform for analysis of movement^69,70^. Before heat shock, mutants of *gnrr-6* behaved like animals with a wild-type background (Fig. 4C). However, loss of *gnrr-6* attenuated the somnogenic effect of *nlp-22* overexpression on locomotion (Fig. 4C). We conclude that GNRR-6, but not GNRR-3, is a receptor for NLP-22 in the regulation of body movement. This conclusion is supported by our *in vitro* data (Fig. 2B) showing that NLP-22 is a potent ligand for GNRR-6, but activates GNRR-3 only at physiologically irrelevant concentrations.

Translational reporter transgenes for *gnrr-6* revealed expression of this gene in neurons involved in locomotory control. Expression of *gnrr-6* localized to SIA sublateral motor neurons and AVB forward command interneurons, which is in agreement with single-cell RNA-Seq data^71^. In addition, we observed expression in PDB and PHC neurons in the tail and few sensory neurons in the head (Supplementary Fig. S6a–h). Available single-cell RNA-Seq data suggests that additional neurons, including OLL, URB and AWC neurons, may express *gnrr-6*^71^. Expression of *gnrr-3* was observed in several inhibitory GABAergic motor neurons of the ventral nerve cord (VNC) in the distal tail (Supplementary Fig. S6i,j). These distinct expression patterns suggest that GNRR-3 and GNRR-6 act in different locomotory circuits, which is in line with our finding that NLP-22 affects locomotion quiescence through GNRR-6 but not GNRR-3.

### NLP-2 RPamide neuropeptides reduce locomotion quiescence during L4 lethargus

**Figure 5:**
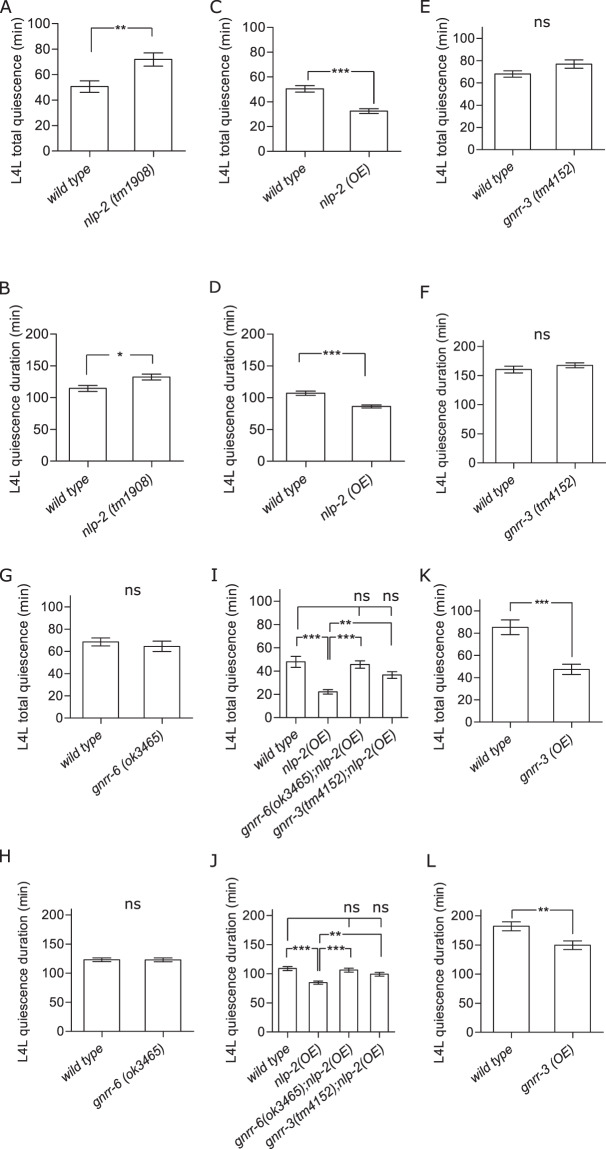
GNRR-3 and GNRR-6 are required for the wake-promoting effects of *nlp-2* overexpression. Average total quiescence during L4 lethargus (L4L) and average quiescence duration of L4L for (**A**,**B**) *nlp-2* mutants (N ≥ 20 animals) and (**C**,**D**) animals overexpressing *nlp-2* from an *nlp-2p::nlp-2* transgene (N > 27 animals). (**E**,**F**) Mutants for *gnrr-3* (N > 15 animals) and (**G**,**H**) g*nrr-6* (N > 21 animals) are not defective in lethargus quiescence. (**I**,**J**) Disrupting *gnrr-3* or *gnrr-6* abolishes locomotory quiescence in animals overexpressing *nlp-2* (N ≥ 31 animals). (**K**,**L**) Animals overexpressing *gnrr-3* show increased movement quiescence during L4L (N = 18 animals). Student’s two-tailed t-tests or One-way ANOVA and Tukey test; ***P < 0.001; **P < 0.01; *P < 0.05; ns, not significant (*P* > 0.05); error bars represent SEM.

Since NLP-2 and NLP-23 neuropeptides activated the same receptors *in vitro* as NLP-22, we asked whether genetically manipulating genes encoding these neuropeptides affects locomotion quiescence. We measured total movement quiescence and quiescence duration during L4 lethargus of *nlp-2* and *nlp-23* loss-of-function mutants. *nlp-23* mutants displayed no difference in quiescence compared to wild-type animals (Supplementary Fig. S7). By contrast, *nlp-2* mutants showed increased movement quiescence and quiescence duration during L4 lethargus (Fig. 5A,B). The opposite phenotypes, a decrease in total quiescence and quiescence duration (Fig. 5C,D), were induced by multi-copy overexpression of *nlp-2* from its endogenous promoter. These data suggest that NLP-2 peptides promote wakefulness during L4 lethargus. In adult animals, both *nlp-2* overexpression and loss-of-function reduced locomotion activity (Supplementary Fig. S8), suggesting that concentrations of NLP-2 peptides below or above physiological levels alter locomotion differently during adulthood and lethargus. Although *nlp-2* derived peptides activate the same receptors *in vitro* as the somnogenic NLP-22 neuropeptides, our *in vivo* experiments suggest that NLP-2 neuropeptides promote movement rather than quiescence during lethargus.

### GNRR-3 and GNRR-6 are required for wake-promoting effects of *nlp-2* overexpression

If NLP-2 peptides were signaling through GNRR-3 and/or GNRR-6, then loss of these receptors’ functions may have the same phenotype as *nlp-2* loss-of-function. Total quiescence and quiescence duration during L4 lethargus in *gnrr-3* and *gnrr-6* mutants were not different from wild-type controls (Fig. 5E–H). Since overexpression of *nlp-2* decreased behavioral quiescence (Fig. 5C,D), an effect opposite to that of NLP-22/GNRR-6 signaling, we hypothesized that NLP-2 signals through a different receptor than NLP-22. Our *in vitro* data indicated that NLP-2 neuropeptides are potent ligands of both GNRR-3 and GNRR-6 (Fig. 2A), in contrast to NLP-22 which signals via GNRR-6 and not via GNRR-3. If GNRR-3 or GNRR-6 is a receptor for NLP-2 in regulating quiescence, then *gnrr-3* and/or *gnrr-6* loss-of-function should abrogate the wake-promoting effects of *nlp-2* overexpression. We found that disrupting either *gnrr-3* or *gnrr-6* abolished the reduced quiescence in animals overexpressing *nlp-2* (Fig. 5I,J). Thus, both *gnrr-3* and *gnrr-6* are required for the wake-promoting effects of *nlp-2* overexpression during lethargus.

Our behavioral data suggests that the RPamide receptor GNRR-6 is required for the regulation of lethargus quiescence by both NLP-2 and NLP-22 neuropeptides, whereas NLP-2/GNRR-3 signaling is additionally required in order to increase wakefulness rather than quiescence. As these receptors seem to be expressed in non-overlapping subsets of neurons (Supplementary Fig. S6), we asked if overexpression of *gnrr-3* alone is sufficient to decrease lethargus quiescence. Overexpression of *gnrr-3* indeed decreased total quiescence and quiescence duration during L4 lethargus (Fig. 5K,L). Thus, overexpression of *nlp-2* and *gnrr-3* result in similar wake-promoting phenotypes. This effect on movement was restricted to lethargus as adult worms that lacked *gnrr-3* or that overexpressed *gnrr-3* did not show altered locomotory activity (Supplementary Fig. S8). In sum, GNRR-6 signaling is required for the RPamide-mediated regulation of movement during lethargus, while NLP-2/GNRR-3 signaling is additionally required to mediate *nlp-2*-induced wakefulness rather than quiescence.

### NLP-2 peptides do not modulate feeding quiescence and sensory arousal threshold during L4 lethargus

Behavior during lethargus is characterized by locomotion quiescence, feeding quiescence, and reduced responsiveness to external stimuli^2,31^. To assess whether NLP-2 signaling affects feeding quiescence, we analyzed the duration of feeding quiescence during L4 lethargus for *nlp-2* mutants and for animals overexpressing *nlp-2*. There was no difference in the duration of feeding quiescence, indicating that NLP-2 signaling controls movement quiescence but not feeding quiescence (Supplementary Fig. S9a).

Other mutants with reduced quiescence during lethargus, such as *egl-4* and *npr-1* mutants, show increased responsiveness to sensory stimuli during lethargus^2,21,31^, possibly explaining their arousal phenotype. To test whether the reduction of movement quiescence can be explained by an increased sensitivity to arousing stimuli, we measured the latency required for animals to be aroused by blue light during lethargus. There was no significant difference in response latency between wild type worms and animals lacking or overexpressing *nlp-2* (Supplementary Fig. S9b). Thus, the reduced quiescence phenotype of worms overexpressing *nlp-2* appears specific for movement quiescence, although increased sensitivity to other sensory cues (like chemicals or touch) cannot be excluded.

### *nlp-2* expression cycles with a developmental clock

**Figure 6:**
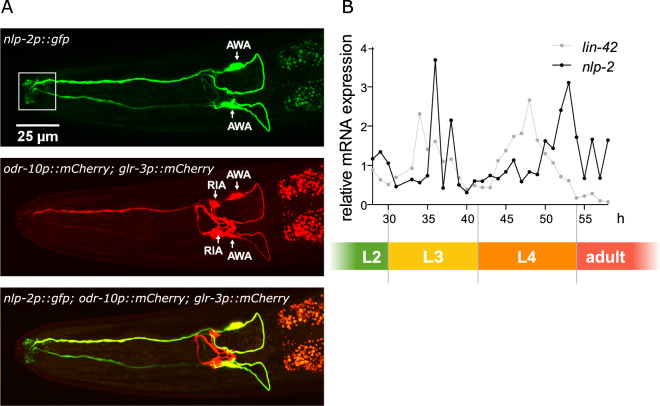
Expression of *nlp-2* localizes to AWA neurons and cycles with larval periodicity. (**A**) Expression pattern of a transcriptional *nlp-2* reporter transgene *[nlp-2p::gfp]* in the head region. The upper panel shows the green fluorescent channel displaying *[nlp-2p::gfp]* transgene expression. The middle panel shows the red fluorescent channel with expression of two marker transgenes: *[odr-10p::mCherry]* in AWA and *[glr-3p::mCherry]* in RIA. The lower panel shows an overlay between the green and red channels, demonstrating that the expression of *[nlp-2p::gfp]* colocalizes with the AWA marker construct *[odr-10p::mCherry]*, but not with the *[glr-3p::mCherry]*. The white box in the upper panel indicates the characteristic cilia at the dendrite tips of the AWA amphid sensory neurons expressing GFP. (**B**) Relative qRT PCR expression of *nlp-2* during larval development. The *nlp-2* and *lin-42* expression levels are plotted during one-hour time intervals of postembryonic development at 20 °C after L1 larval arrest. Larval stage indications are based on the complete temporal *lin-42* expression profile (ranging from 0 to 75 hours after hatching).

To identify the cells that express *nlp-2*, we generated a transcriptional green fluorescent protein (GFP) reporter construct. Expression of the *nlp-2p::gfp* reporter transgene was restricted to one pair of head neurons and four uterine cells. Based on their location and sensory cilia morphology, we identified the head neurons as the olfactory AWA neurons (Fig. 6A). The uterus cells were identified as the neurosecretory uv1 cells^72^.

The somnogenic RPamide NLP-22 has a cyclical mRNA expression pattern concurrent with peaks in the mid larval stages prior to lethargus^41^. Therefore, we investigated whether the expression pattern of *nlp-2* mRNA also cycles throughout development. We used quantitative reverse-transcription PCR (qRT-PCR) to analyze *nlp-2* mRNA expression over a 30 h time frame, which covered both L3 and L4 lethargus periods. Developmental progression was timed by the transcript profiles of *lin-42*, the *C. elegans* ortholog of the core circadian regulator PERIOD. Similar to PERIOD, which shows cyclic expression with a circadian periodicity in mammals and insects^12,73^, *lin-42* transcript levels cycle with *C. elegans* larval stages, being lowest during each molt^13^. We found that *nlp-2* expression cycles with a constant phase relationship to *lin-42* during larval development (Fig. 6B). *nlp-2* mRNA expression peaked in preparation of the L3 and L4 molts, when *lin-42* levels are low, suggesting that *nlp-2* expression is regulated, at least partially, at the transcriptional level.

## Discussion

Sleep, wakefulness and the transition between these behavioral states are regulated by the coordinated interplay of neuronal circuits in which neuropeptide signaling plays an essential role^19,23,74^. Neuropeptides, such as mammalian hypocretin/orexin and melanin-concentrating hormone, can have arousing or somnogenic effects, respectively^22,75,76^, suggesting that the balanced action of sleep- and wake-promoting neuropeptides is a conserved mechanism for regulating sleep/wake cycles.

Here, we provide evidence for two GnRH-like neuropeptidergic systems promoting sleep and wakefulness in *C. elegans*. NLP-2 RPamide signaling impairs movement quiescence during lethargus, which is opposite to the effect of the somnogenic NLP-22 RPamide neuropeptide that induces behavioral quiescence. RPamide neuropeptides – comprising *nlp-2*, *nlp-22*, *nlp-23* and *nlp-46* encoded peptides – are highly conserved in nematodes and share subtle sequence similarities to members of the bilaterian GnRH/AKH peptide family. GnRH-like signaling displays urbilaterian conservation and has well-established roles in reproductive maturation and behavior as well as in energy homeostasis^52–56,77–81^.

To date, a direct role for GnRH/AKH systems in the regulation of sleep and wakefulness has been described only in *D. melanogaster*, where neuronal AKH/AKHR signaling is required for starvation-induced sleep suppression^45,82^. While *Drosophila* uses hyperactivity as a survival strategy to prevent starvation, *C. elegans* larvae respond to extended starvation by increased sleep and developmental arrest to prolong healthy lifespan^83^. In adult *C. elegans*, however, food deprivation also leads to suppression of heat stress-induced quiescence and this suppression is increased with population density^84^.

Our results suggest a model in which the RPamide neuropeptides NLP-2 and NLP-22 signal through GNRR-3 and GNRR-6 with opposing effects on locomotory quiescence during lethargus. Consistent with a neuropeptide system sufficient for promoting movement, we found that overexpression of either *nlp-2* or *gnrr-3* reduces quiescence during L4 lethargus. Although overexpression of a peptide may result in levels higher than those normally present *in vivo*, *nlp-2*-induced wakefulness during L4 lethargus requires both *gnrr-3* and *gnrr-6*. This finding suggests that NLP-2 neuropeptides signal through these receptors *in vivo* and is in agreement with our cell-culture experiments, in which NLP-2 peptides activated both GNRR-3 and GNRR-6. By contrast, the somnogenic NLP-22 peptide increases quiescence by signaling via GNRR-6 but not via GNRR-3. Taken together, these results suggest that GNRR-6 is required for the regulation of lethargus quiescence by RPamide neuropeptides. We propose that NLP-22 activates GNRR-6, but not GNRR-3, resulting in quiescence, whereas NLP-2 peptides additionally activate GNRR-3, which promotes wakefulness rather than sleep. As *gnrr-3* and *gnrr-6* seem to be expressed in non-overlapping subsets of neurons, NLP-2/GNRR-3 signaling may indirectly interfere with NLP-22 pathways, although the mechanisms underlying such interactions remain unclear. While disrupting *nlp-2* or *nlp-22* affects quiescence during lethargus, *gnrr-3* and *gnrr-6* mutants display normal lethargus, which might be explained by additional as yet unidentified RPamide receptors.

The observation that both GNRR-3 and GNRR-6 elicit a cellular calcium response *in vitro* without Gα_16_ suggests signaling via Gα_q_, which is in agreement with a previous study reporting that Gα_q_ signaling controls sleep/wake-like states in *C. elegans*^85^. The role of NLP-2, GNRR-3 and GNRR-6 in behavioral quiescence seems restricted to locomotion, as neither feeding quiescence, nor *nlp-22*–induced pharyngeal pumping quiescence is affected in animals with altered *nlp-2* expression levels or lacking these receptor systems, respectively. Disrupting NLP-2 signaling also leads to the preservation of a normal threshold for sensory arousal, in contrast to other neuropeptidergic systems, like NPR-1 and its ligands FLP-18 and FLP-21, that stimulate both sensory and locomotory activity during lethargus^86^.

Our results suggest that lethargus in *C. elegans* is regulated by the balanced and cyclic action of sleep- and wake-promoting neuropeptides. Signaling by NLP-2 neuropeptides, like NLP-22^41^, is at least partially regulated at the level of mRNA transcripts, which cycle relative to a LIN-42/PERIOD-based larval clock that controls the synchronization of lethargus quiescence^13^. Peak expression of *nlp-2* is delayed compared to the expression of the *lin-42* gene, the *C. elegans* ortholog of the circadian clock gene *period*, which sets the timing for sleep-like behavior. This observation is in line with our evidence for the wake-promoting effects of NLP-2. How might the cyclic expression of *nlp-2* be regulated? The upregulation of *nlp-2* transcripts when *lin-42* expression is high suggests that *nlp-2* expression can be a clock output signal, regulated by the activity of LIN-42. Interestingly, a similar mechanism has been described for regulating the expression of *nlp-22*, which oscillates in response to the LIN-42/PERIOD-based larval clock^41^. The *nlp-2* and *nlp-22* genes are clustered on the X chromosome, suggesting a transcriptional co-regulation of these wake- and sleep-promoting signals.

Both literature^41^ and our locomotion quiescence data (Fig. 4) suggest that RPamide peptide concentrations are tightly regulated, as both decreasing them below or increasing them above physiological levels alters locomotion. For *nlp-2*, deviation from this set-point in either direction reduces locomotion in adults, but how this occurs mechanistically remains unclear. We propose that elevated levels of NLP-2 increases locomotion during larval sleep, possibly by acting as a molecular switch to wakefulness via its additional activation of GNRR-3, and that NLP-2 levels are subsequently maintained within a physiological range during normal locomotion in adults.

Expression of *nlp-2* was restricted to a pair of olfactory AWA neurons and vulval uv1 cells, consistent with previously reported expression patterns^87^. The *nlp-2*-expressing AWA neurons have ciliated sensory endings and are known to display pulsatile calcium transients, which are elicited by action potential bursts^88,89^. AWA neurons display sex-specific pheromone responses^90^ and may share some functional similarity to chordate GnRH neurons that arise from the olfactory placode^91,92^ and are also presumed to regulate non-reproductive functions in larval stages^93,94^. Our expression data suggest that NLP-2 neuropeptides from AWA neurons may act on GNRR-3 and GNRR-6 in neurons of the motor circuit to mediate wakefulness. The integration of environmental and intrinsic signals enables the coordination of sleep-wake states with competing and complementary animal behaviors, such as foraging and mating^95,96^. The release of NLP-2 from sensory neurons in response to environmental and/or internal stimuli may therefore contribute to a switch between sleep-wake states.

The somnogenic RPamide NLP-22 is expressed in a different site, the glutamatergic RIA interneurons^41^, which have no direct synaptic connections to AWA sensory neurons, but are also involved in sensorimotor integration and olfactory steering (Supplementary Fig. S10)^97,98^. The NLP-22 receptor GNRR-6 is expressed in sublateral motor neurons and interneurons that project along the ventral nerve cord, which suggests a role in locomotion quiescence. NLP-22-induced feeding quiescence is indeed unaffected in *gnrr-6* mutants, suggesting that NLP-22 signals through an additional thus far unidentified receptor to inhibit feeding during sleep.

Reported GnRH-associated phenotypes together with the data presented here may hint at a conserved role for GnRH/AKH-like signaling in circadian and developmental clock-mediated metabolic and locomotion activity patterns^99^. In mammals, reduced sleep during the proestrus phase, when GnRH pulse frequency increases, suggests a role in wakefulness^100,101^. GnRH-like neuropeptides have also been implicated in the timing of insect pupariation/ecdysis^102^. Like *C. elegans* lethargus, ecdysis is characterized by reduced feeding and locomotion quiescence and eventually leads to sexual maturation. The role of GnRH-like signaling in the cyclic regulation of metabolism and reproduction, such as cyclic larval/juvenile ecdysis, seasonal breeding and estrous cycle, can be reconciled with its role in sleep-wake behavior as coordinating and coupling diverse metabolic cycles to behavioral responses across Bilateria.

## Materials and Methods

### Strains and cultivation

Strains were cultured at 20 °C under standard conditions on NGM agar plates seeded with *Escherichia coli* OP50^103^. The following wild type and mutant strains were used: N2 (Bristol), LSC509 *[gnrr-6 (ok3465) X]* (x2), LSC714 *[gnrr-3 (tm4152) X]* (x8), FX01908 *[nlp-2 (tm1908) X]* and NQ638 *[nlp-23 (tm5531) X]* (x2) (x# indicated times outcrossed to N2). Transgenic strains used in this study are listed in Supplementary Table S1.

### Phylogenetic analysis

For the phylogenetic analysis of GnRH/AKH-like receptors, the protein dataset was composed of deuterostomian GnRH receptors, bilaterian corazonin (Crz) receptors, protostomian GnRH/AKH receptors and nematode GnRH/AKH-like receptors. Arginine vasopressin (AVP) and neuropeptide S (NPS) receptor sequences were used as outgroup. Accession numbers of the sequences are listed in Supplementary Table S2. Sequence alignments were generated using the Simultaneous Alignment and Tree Estimation (SATé) software package, which uses an iterative greedy search heuristic to sequentially align sequences and compute a maximum likelihood phylogenetic tree from alignments^104^. The final maximum likelihood phylogeny was estimated using PhyML. The following parameters were used: LG as the amino-acid replacement matrix^105^, Subtree Pruning and Regrafting (SPR) and Nearest Neighbor Interchange (NNI) for topological moves^106^, and a number of discrete gamma rate categories equal to 4. Branch support values were generated using nonparametric bootstrapping (100 bootstraps). Branches with bootstrap values below 50% were collapsed.

Peptide sequence alignments in Supplementary Fig. S5 (A,B) were generated using the MUSCLE algorithm in MEGA 7. Panel C was first aligned with the MUSCLE algorithm and afterwards adjusted manually into separated boxes of similar sequences to avoid larger gaps in the multiple sequence alignment, though predicted color coding was maintained. Full species names and Genbank sequence accession numbers are listed in Supplementary Table S3.

### Molecular biology

For receptor deorphanization, the open reading frame of each receptor was cloned into the pcDNA3.1D/V5-His TOPO mammalian expression vector. Only receptors with a seven alpha-helical transmembrane topology, predicted using TMHMM 2.0 software, were cloned^107^. Sequences of receptor cDNAs (GNRR-1a, GNRR-2a, GNRR-3, GNRR-5, GNRR-6, GNRR-7 and DAF-38/GNRR-8) were verified to yield identical protein sequences as the corresponding translated cDNA sequences on WormBase (WS235). Expression plasmids were isolated for transfection of mammalian cells using the EndoFree Plasmid Maxi Kit (Qiagen).

For the *nlp-22* heat shock-inducible overexpression strains, *gnrr-3* and *gnrr-6* mutants were crossed with NQ251 carrying a [*hsp16.2p:nlp-22; hsp16.2p::gfp; myo-2p::mCherry*] transgene^41^. For *nlp-2* and *gnrr-3* overexpression, a linear *nlp-2p::nlp-2* and *gnrr-3p::gnrr-3* PCR construct was amplified from wild type *C. elegans* genomic DNA using Herculase Enhanced DNA polymerase (Agilent Technologies). Primers used for PCR amplification are listed in Supplementary Table S4.

Transcriptional GFP reporter constructs for *nlp-2* and *gnrr-3* were created using overlap-extension PCR as described^108^. A translational GFP reporter construct for *gnrr-6* was PCR amplified from a commercially available fosmid vector (TransgeneOme clone 9914866399944241 D12; Source BioScience). A translational *gnrr-6p::gnrr-6::SL2-mKate* reporter construct was generated using the Multisite Gateway Three-Fragment cloning system (12537-023, Invitrogen) into pDESTR4R3 II. The respective promoter lengths upstream of the predicted start codon used for *nlp-2* and *gnrr-3* transcriptional reporter constructs were 2062 bp and 1877 bp. The translational reporter construct for *gnrr-6* consisted of 2960 bp promoter sequence, the *gnrr-6* coding sequence without stop codon, and a *gfp* sequence inserted 66 bp after the *gnrr-6* coding sequence or an SL2::mKate sequence. Primers used for PCR amplification are listed in Supplementary Table S5.

### Transgenesis

Transgenic worms were created by microinjection using a Leica DMIRB inverted DIC microscope equipped with an Eppendorf Femtojet microinjection system. Each construct was injected at a concentration of 50 ng/µl together with 1 kb DNA ladder as carrier DNA and 5 ng/µl pCFJ90 (*myo-2p::mCherry*) or a combination of 5 ng/µl *rol-6p::rol-6(d)* and *glr-3p::mCherry* as co-injection marker.

### Peptide synthesis and purification

Peptides were custom-synthesized by GL Biochem Ltd. All peptides were initially tested at a concentration of 10 µM. Receptor activating peptides were purified using reverse-phase HPLC and verified using MALDI TOF mass spectrometry. Peptide concentrations were determined with a bicinchoninic acid (BCA) assay^109^. For receptor activation assays, peptides were first lyophilized and then diluted to the desired concentrations.

### Receptor activation assay

Chinese hamster ovary (CHO) cells stably expressing apo-aequorin and the promiscuous Gα_16_ subunit were used for receptor deorphanization (ES-000-A24, Perkin-Elmer). To characterize downstream signaling, CHO cells stably expressing apo-aequorin but lacking the promiscuous Gα_16_ protein were used (ES-000-A12, Perkin-Elmer). Cells were cultured in Dulbecco’s Modified Eagle’s Medium Nutrient Mixture Ham F-12 (DMEM/F-12, Invitrogen) to which 1% penicillin/streptomycin, 2.5 µg/ml amphotericin B, and 10% fetal bovine serum (Sigma-Aldrich) were added. Growth medium was supplemented with 250 µg/ml zeocin or 5 µg/ml puromycin, which serves as a selection reagent for CHO cells with or without the promiscuous Gα_16_ subunit, respectively. Cells were grown as a monolayer at 37 °C, 5% CO_2_ and high humidity. For transfection, 3.75 ml Opti-MEM I (Invitrogen), 7.5 µg pcDNA3.1 construct and 18.75 µl Plus reagent (Invitrogen) were gently mixed in a polystyrene tube. After incubation for 5 minutes at room temperature, 45 µl Lipofectamine LTX (Invitrogen) was added and gently mixed. The transfection reagent was incubated for 30 min at room temperature. Growth medium was removed leaving 3 ml and the transfection reagents were added dropwise to the cells. Transfected cells were grown overnight and 20 ml growth medium was added the next day. Cells were grown one more day before the assay at 28 °C.

Two days after transfection, CHO cells were detached from the surface of the culture flask using phosphate buffered saline with 0.2% EDTA and collected in 10 ml colorless DMEM/F-12 (11039, Gibco). Cell viability was measured using a NucleoCounter NC-100 (Chemometic). Cells were pelleted for 4 min at 800 rpm at room temperature and resuspended to a concentration of 5 × 10^6^ cells/ml in colorless DMEM/F12 with 0.1% bovine serum albumin (BSA). 5 µM coelenterazine H (Invitrogen) was added to the cell suspension. Cells were incubated by gentle shaking for 4 hours in the dark at room temperature, allowing the aequorin holoenzyme to be reconstituted. After a 10-fold dilution in DMEM/F12 with 0.1% BSA, the cells were incubated for another 30 min. Peptides were dissolved in DMEM/F12 with 0.1% BSA and 50 µl of the peptide solution was added to the wells of a white flat bottom 96-well plate. Wells containing DMEM/F12 with 0.1% BSA were used as a negative control, while wells containing 1 µM ATP were used as a positive control. Incubated cells were added to the wells at a density of 25,000 cells/well and luminescence was monitored for 30 s on a Mithras LB 940 luminometer (Berthold Technologies). After 30 seconds, 0.2% Triton X-100 dissolved in DMEM/F12 with 0.1% BSA was added to lyse the cells and light emission was recorded for another 8 seconds. Light emission from each well was calculated relative to the total calcium response (ligand + Triton X-100). EC_50_ values were calculated from dose-response curves that were constructed using a nonlinear regression analysis with a sigmoidal dose-response equation (Graphpad Prism 5).

### Fluorescence microscopy

Transgenic reporter animals were mounted on 2% agarose pads and immobilized with 5 mM sodium azide. Fluorescence was observed on an Olympus Fluoview FV1000 (IX81) confocal microscope. Confocal Z-stack images were processed using Imaris 7.2 (Olympus).

The *nlp-2p::gfp* localization construct was co-injected with a red fluorescent *glr-3p::mCherry* marker construct that cell-specifically expresses in the RIA neurons and a *rol-6* dominant *roller* co-injection marker. The resulting NQ744 *qnEx423 [nlp-2p::gfp; glr-3p::mCherry; rol-6]* strain was then crossed with LSC1298 *lstEx682 [odr-10p::mCherry::3*′*UTR odr-10; unc-122p::gfp]* to colocalize its expression in AWA.

To identify expression in amphid sensory neurons, LSC1687 *lstEx1023 [gnrr-6p::gnrr-6::gfp; unc-122p::mCherry]* animals were stained with DiI. Similarly, a second *gnrr-6* reporter strain LSC1904 *lstEx1048 [gnrr-6p::gnrr-6::SL2::mKate; unc-122p::gfp]* was stained with DiO to confirm expression of *gnrr-6* in ASK. To colocalize transgene expression or to exclude *gnrr-6* expression in specific neurons, LSC1904 was also crossed with the following marker strains: for PHC neurons BL5717 *inIs179 [ida-1p::gfp] II; him-8(e1489) I*, for AVB neurons AQ2529 *ljEx286 [sra-11p::YC3.60]*, for SMB neurons AQ3642 *ynIs25 [flp-12p::gfp; rol-6d]*, for SMD neurons AQ3848 *kyIs123 [trp-1p::gfp]*, and for glutamatergic neurons OH12312 *otIs388 [eat-4(fosmid)::SL2::yfp::H2B; pha-1(e2123)]; him 5(e1490)*. GFP-positive cells in LSC1091 *lstEx556 [gnrr-3p::gnrr-3::gfp; unc-122p::mCherry]* were identified by crossing with a red fluorescent GABAergic reporter strain, XE1375 *wpIs36 [unc-47p::mCherry]*.

### Developmental time course of mRNA expression

Developmental mRNA expression was analyzed using qRT-PCR as described^110^. Wild type *C. elegans* were synchronized as L1 diapause larvae and cultured in S-medium^103^ with E. *coli* K12 as food source, while gently shaking at 20 °C. Worms were sampled every hour. mRNA was isolated (Rneasy Mini kit, Qiagen) and reverse transcribed to cDNA (SuperScript III Reverse Transcriptase, Invitrogen) using random primers (Invitrogen). Primer pairs for *nlp-2* were designed with Primer Express (Applied Biosystems) and VectorNTI (Invitrogen). The specific primers used for qPCR of *nlp-2* transcripts were: forward 5′-CTGAAGGAGCAATGGGCAAA −3′ and reverse 5′-ATGATGAGATCACTAACATCCACAG −3′. The transcript profile of *lin-42b/c* was used as a marker for developmental timing^13^, using *lin-42 fwd* TGTGCCCAACGCCAATC and *lin-42 rev* CACCTTCCTCACGCATTGC. A melt curve analysis confirmed the absence of primer dimers and other non-specific products. Fast SYBR Green Master Mix (Applied Biosystems) was used for qRT-PCR and performed using the StepOnePlus Real-Time PCR system (Applied Biosystems). Cycling parameters were 600 s at 95 °C, followed by 40 cycles of 3 s at 95 °C and 30 seconds at 60 °C. Each sample was analyzed in triplicate to assess technical variation. A no template control consisting of milli-Q water instead of cDNA was added as a negative control. The normalized relative quantity of cDNA was calculated using the geometric mean of three reference genes (*cdc-42*, *tba-1* and *pmp-3* as the three best performing out of *cdc-42*, *tba-1, pmp-3, rpb-12, gpd-2* and *Y45F10D.4* using geNorm^111^).

### Behavioral assays

Measurements of feeding and locomotion quiescence after heat-shock induced expression of *nlp-22* (Fig. 4A,B) were performed according to Nelson *et al*.^41^. Day one adult worms were placed on a 55 mm diameter NGM agar plate seeded with *E. coli* OP50. Plates were double wrapped with parafilm and incubated in a water bath at 33 °C for 30 min. After heat-shock, worms were recovered at 20 °C for 2–3 hrs. To quantify feeding quiescence, pharyngeal pumps were counted for 20 s. A pump was counted as one complete phase of contraction and relaxation, based on the anterior-posterior movement of the grinder in the terminal bulb. This was done at 80X on a stereomicroscope. For locomotion quiescence, body bends were manually counted for 1-minute time intervals. A bend was counted as a single turn (i.e. half phase) in either direction during normal forward movement. This was done at 40–80X on a stereomicroscope. Long-term behavioral tracking of locomotory quiescence pre- and post-heat shock (Fig. 4C) was measured with the WorMotel system as described below.

For measurements of total quiescence and quiescence duration during L4 lethargus, worms were monitored beginning in the L4 stage for 9 hrs in 2 concave wells (3 mm diameter, 2.5 mm depth) of a polydimethylsiloxane (PDMS) chip filled with 15 µl NGM agar and seeded with *E. coli* OP50^112^. For each measurement, one control and one experimental animal were manually placed in adjacent wells. The PDMS chip was placed on a microscope base (Diagnostics Instruments) with a fiber optic cable DCR III light source (Schott) for bright-field illumination. Worms were monitored by a camera (659 × 494 pixels, scA640–70fm, Basler Vision Technologies) which was mounted on a stereomicroscope (Zeiss Stemi 2000). 8-bit grayscale images with a spatial resolution of 12.5 µm per pixel were captured every 10 s. The quiescence parameters “total quiescence” and “quiescence duration” are defined as in Raizen *et al*.^2^. Quiescence was quantified using a machine vision frame subtraction method^2^ and statistically compared to wild-type control animals with paired t-tests. All quiescence experiments using this method (Fig. 5E,F,K,L and Supplementary Fig. S7) were done in a temperature-controlled room at 20 °C.

Locomotion quiescence during L4 lethargus (Fig. 5A–D,G–J) and adult locomotion activity (Fig. 4C) was also quantified using a medium-throughput WorMotel system. WorMotel analyses were conducted as described previously^69^. Briefly, 24-wells of a polydimethylsiloxane (PDMS) chip (gifts from Chris Fang-Yen, University of Pennsylvania) were filled with NGM/agar and allowed to cool to room temperature. L4 animals were identified to be pre-lethargus due to their active feeding behavior (i.e. pharyngeal pumping) and transferred to a freshly seeded plate. Moving them to a plate prior to the WorMotel prevented the accidental transfer of eggs and other larvae. Individual active L4 animals were then transferred to the surface of the agar in the 24-welled chip. A small amount of DA837 bacteria was transferred with the animal at this time, using a worm pick. The chip was placed in a petri-dish, which was sealed with parafilm and transferred into the WorMotel imaging system. Images were taken every 10 seconds for 12 hours. Using published MatLab software^69^, pixel subtraction followed by quiescence analyses were conducted to produce the total amount of quiescence every 10-minutes during the 12-hour period. Lethargus periods were manually identified based on an identifiable 1–2-hour peak of quiescence, which usually occurred within the first 2–4 hours of imaging. If a peak was not detected because of high background, the images were manually observed for the absence or death of an animal, and these data point were censured. We also censured data in which the animals appeared to fall asleep during the preparation of the chip. This was evident by the peak of quiescence beginning immediately after the start of the recording. WorMotel assays were performed at temperatures ranging from 22.5 to 24 °C. Quiescence was statistically compared to wild type control animals with unpaired t-tests. Statistical analysis was always performed with internal wild-type controls.

To measure the duration of L4 lethargus feeding quiescence (Supplementary Fig. S9a), late L4 worms, which had not yet entered lethargus, were individually transferred to freshly seeded NGM agar plates. Pharyngeal pumping was observed by stereomicroscopy every 10 min. Quiescence duration was measured as the time between the offset and onset of pharyngeal pumping.

For adult locomotion assays (Supplementary Fig. S8), synchronized day 1 adult animals were imaged for 10 min while moving on fresh NGM plates at 20 °C that were seeded 24 hrs in advance with 200 µl of OP50 bacterial culture. High-resolution acquisition (56 pixels/mm) was performed with a 10 megapixel camera (GigE PRO GP11004M NET 1/2,3″ CMOS 3840 × 2748; with matching lenses LM16JC10M Mp KOWA 2/3″ F1.8) running at 2 frames per second. Animal tracking was achieved with a custom written MATLAB (MathWorks) script^113^. Background subtracted and denoised image frames were binarized to obtain worm shapes in each frame. Shape centroid tracks over time were quality controlled for collisions and smoothed by a rectangular sliding window of 3 centroid positions. The absolute speed was determined as the distance between consecutive centroid positions. Only speed values assigned as forward locomotion runs were averaged for each track. Each experimental day contained an internal wild type control to which other strains were normalized.

Arousal threshold was analyzed by measuring the response latency of individual worms to blue light during lethargus (Supplementary Fig. S9b). A response to blue light was defined as a backward movement equal to one-half of the worm’s length^35^.

### Statistical analysis

Dose-response curves were constructed using a nonlinear regression analysis with a sigmoidal dose-response equation (GraphPad Prism 5). Statistical significance of behavioral assays was determined using (un)paired Student t-tests or one-way ANOVA and Tukey post-hoc for multiple comparisons (as indicated in each figure legend) with the GraphPad Prism version 5 software package. In graphs, error bars represent standard error of the mean (SEM) and significance levels are indicated as: ***P < 0.001; **P < 0.01; *P < 0.05; ns (= not significant) *P* > 0.05. Experiments were performed on at least two independent days.

## Supplementary information

Supplementary information.
