# Supplementary material for: RPamide neuropeptides NLP-22 and NLP-2 act through GnRH-like receptors to promote sleep and wakefulness in C. elegans
Source: Sci Rep. 2020 Jun 18;10:9929. doi: 10.1038/s41598-020-66536-2 (PMC7303124; doi:10.1038/s41598-020-66536-2)
Supplement: Supplementary file 1 — Supplementary information. [file 41598_2020_66536_MOESM1_ESM.pdf]

Supplementary information

RPamide neuropeptides NLP-22 and NLP-2 act through GnRH-like receptors to promote sleep and wakefulness in *C. elegans*

P. Van der Auwera[§], L. Frooninckx[§], K. Buscemi, R. T. Vance, M. D. Nelson, J. Watteyne, O. Mirabeau, L. Temmerman, W. De Haes, L. Fancsalszky, A. Gottschalk, D. M. Raizen, L. Schoofs[*¶], I. Beets[*¶]

[§,¶] These authors contributed equally.
* Corresponding authors

Supplementary Figures S1-S10: p1-12

Supplementary Tables S1-S5: p13-16

References: p17

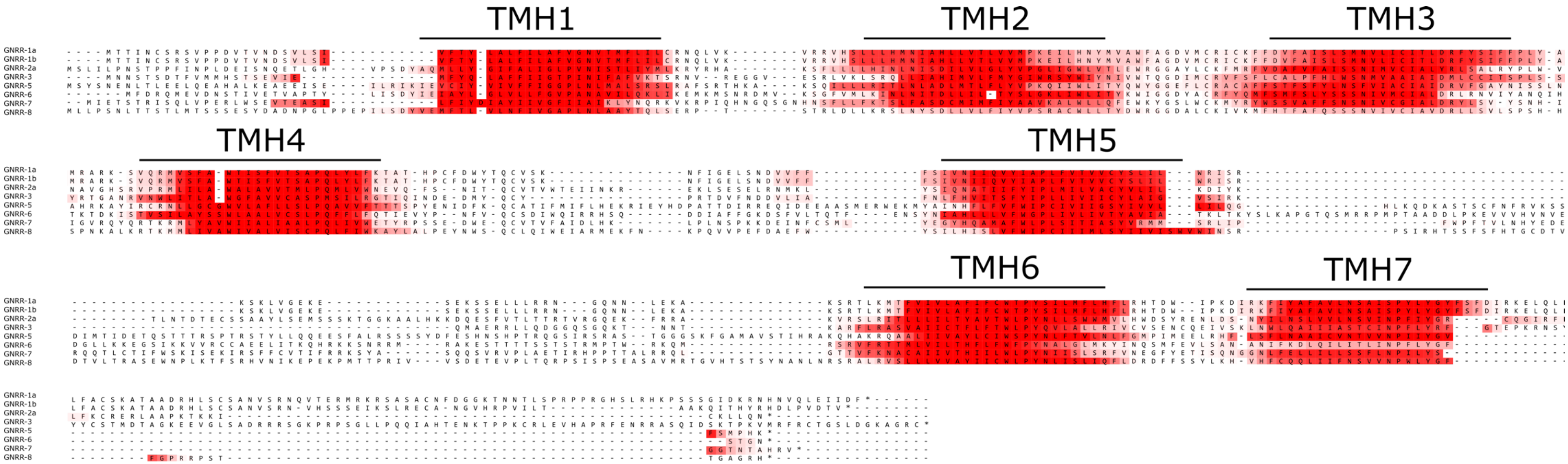

Supplementary Fig. S1. Amino acid sequence alignment of *C. elegans* GNRRs displaying predicted transmembrane helices

Amino acid sequences of *C. elegans* GnRH/AKH-like receptors (GNRR-1 to -3, GNRR-5 to -7 and DAF-38/GNRR-8) were aligned by the MUSCLE algorithm. Red color-coding indicates the transmembrane helix (TMH) domains predicted by the TMHMM tool [1–3].

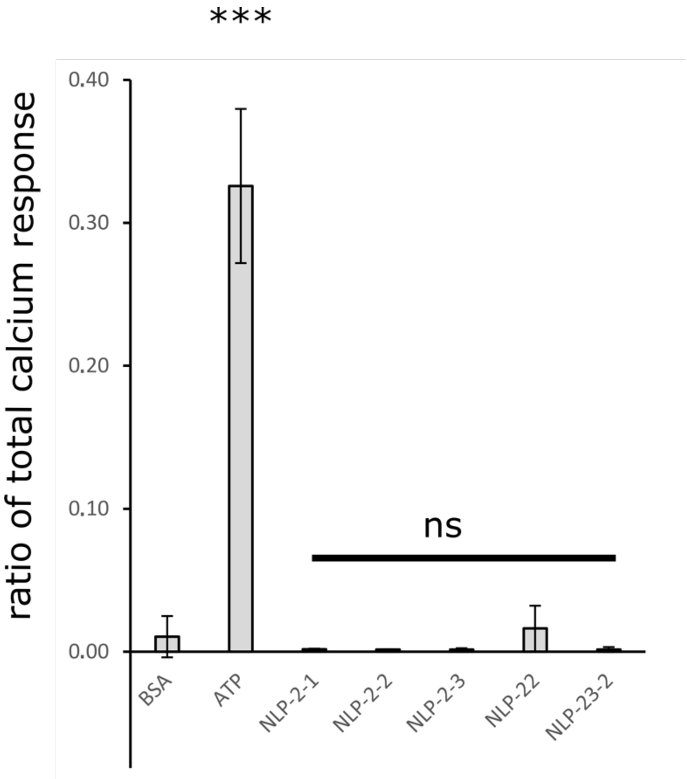

Supplementary Fig. S2. RPamide neuropeptides do not evoke calcium responses in CHO cells transfected with empty vector

RPamide neuropeptides do not elicit a significant Ca^{2+} response in CHO cells transfected with empty pcDNA3.1 vector in comparison to a bovine serum albumin (BSA) control measurement (N > 4). ATP was used as a positive control activating receptors endogenously expressed in CHO cells. Error bars indicate standard deviation (STDEV). One-way ANOVA and Tukey test; ***$P < 0.001$; ns, not significant ($P > 0.05$).

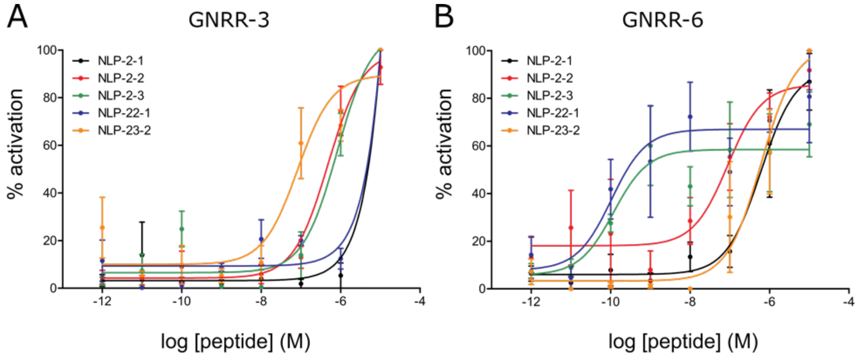

C

Peptide	AA sequence	GNRR-3 EC50	GNRR-3 95% Confidence interval	GNRR-6 EC50	GNRR-6 95% Confidence interval
**NLP-2-1**	SIALGRSGFRPamide	7.3 mM	NA	600.1 nM	183.1 nM to 2.0 μM
**NLP-2-2**	SMAMGRLGLRPamide	478.4 nM	249.7 nM to 916.4 nM	88.6 nM	15.9 nM to 495.2 nM
**NLP-2-3**	SMAYGRQGFRPamide	818.5 nM	395.9 nM to 1.6 μM	118.0 pM	11.0 pM to 1.3 nM
**NLP-22**	SIAIGRAGFRPamide	4.5 mM	NA	104.1 pM	11.4 pM to 947.1 pM
**NLP-23-2**	SMAIGRAGMRPamide	82.4 nM	28.07 nM to 242.4 nM	614.5 nM	184.9 nM to 2.0 μM

Supplementary Fig. S3. GNRR-3 and GNRR-6 activation elicits a cellular calcium response in the absence of Gα_{16}

Dose-response curves of GNRR-3 **(A)** and GNRR-6 **(B)** expressed in CHO cells without a promiscuous Gα_{16} protein are shown as relative (%) to the highest value (100 % activation) after normalization to the total calcium response. Each data point represents the mean ± SEM of N = 4 replicates for each peptide. **(C)** Amino acid (AA) sequences of RPamide neuropeptides activating GNRR-3 and GNRR-6 with their respective mean EC50 values and 95% Confidence intervals.

A

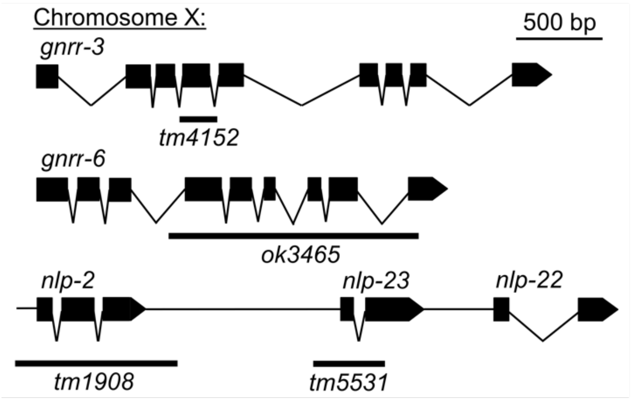

B

```
RefSeq gnrr-6       tcaattttcagATAAGTACAGTATCAATTTTGGCGTACAGTTCTTGGCTTGCTGCACTAGTCT
gnrr-6(ok3465)      tcaattttcagATAAGTACAGTATCAATTTTGGCGTACAGTTCTTGGCTT-------------

GTAGTTTACCACAATTCTTCCTTTTCCAAACAATCGAGGTGTATCCAAATTTTGTTCAATGCTCAGACATTTGGCAAATTCGG
-----------------------------------------------------------------------------------

AGACATAGTCAAGATGATATTGCATTTTTTGGGAAAGACTCGTTTGTACTAACTCAGACATTCGAGAACTCTTACAACATTGC
-----------------------------------------------------------------------------------

TCATTTGgtacatttccaaaaatcttcagtgtcttattaatttttctgtttagCTTCTTGTATTCTGGGGCCCACTGATTGTT
-----------------------------------------------------------------------------------

CTAATCGTAACGTATGCGGTGATAGCAACCAAGCTCACGAAGTACTCGTTAAAAGCACCTGGAACCCAGAGCATGAGAAGACC
-----------------------------------------------------------------------------------

TATGCCAACTGCAGCCGATGATTTACCGAAAGgttcgtatttctaactaccaaaaagaataatagtgaaatctcatatttcca
-----------------------------------------------------------------------------------

gAAGTTGTTGTCCACGTTAACGTCGAAGACGGTCTACTCAAAAAAGAGGGCAGTATAAAAAAGgtggggaacatgcgaaattg
-----------------------------------------------------------------------------------

ataataaaaacagctaaaaattcataaagtacataatctaattatagaacttattacgaactcacatcttgattagttttttt
-----------------------------------------------------------------------------------

ccttgttttctagcaaacttatgaaagaaaatctgtgagatcgagaaagtgaaaacaagctgattactgtagccaatcttcaa
-----------------------------------------------------------------------------------

gttacagGTGGTAAGATGCTGTGCTGAGGAGCTCATTACAAAGCAACATCGAAAGAAGTCGAATCGTAGAATGAGAGCCAgta
-----------------------------------------------------------------------------------

agtgttaaatttttgtgatcacataatgattggatttattaaagAAGAATCAACAACGACAACATCATCAACATCTACACGAA
-----------------------------------------------------------------------------------

TGCCAACGTGGCGGAAACAAATGCGCAGTCGTGTGTTTCGAACCACAATGCTTGTCATTCTGACCCACTTCCTCTTTTGGTTC
-----------------------------------------------------------------------------------

CCTTACAATGCACTCGGCTTAATGAAATACATCAACCAATCTATGTTTGAgtgagtttttgcaaaaaaaa
--------------------AATGAAATACATCAACCAATCTATGTTTGAgtgagtttttgcaaaaaaaa
```

Supplementary Fig. S4. Intron-exon structures and mutant alleles for *gnrr-3, gnrr-6, nlp-2*, *nlp-22* and *nlp-23*

(A) Intron-exon structures of genes with coding sequences depicted as solid boxes. Sequences deleted in alleles *tm4152, ok3465, tm1908 and tm5531* are indicated as bars. (B) The *gnrr-6(ok3465)* allele was sequenced in this study and the 946 bp deletion (indicated by hyphens) aligned to the *gnrr-6* reference sequence. Capital letters indicate exons.

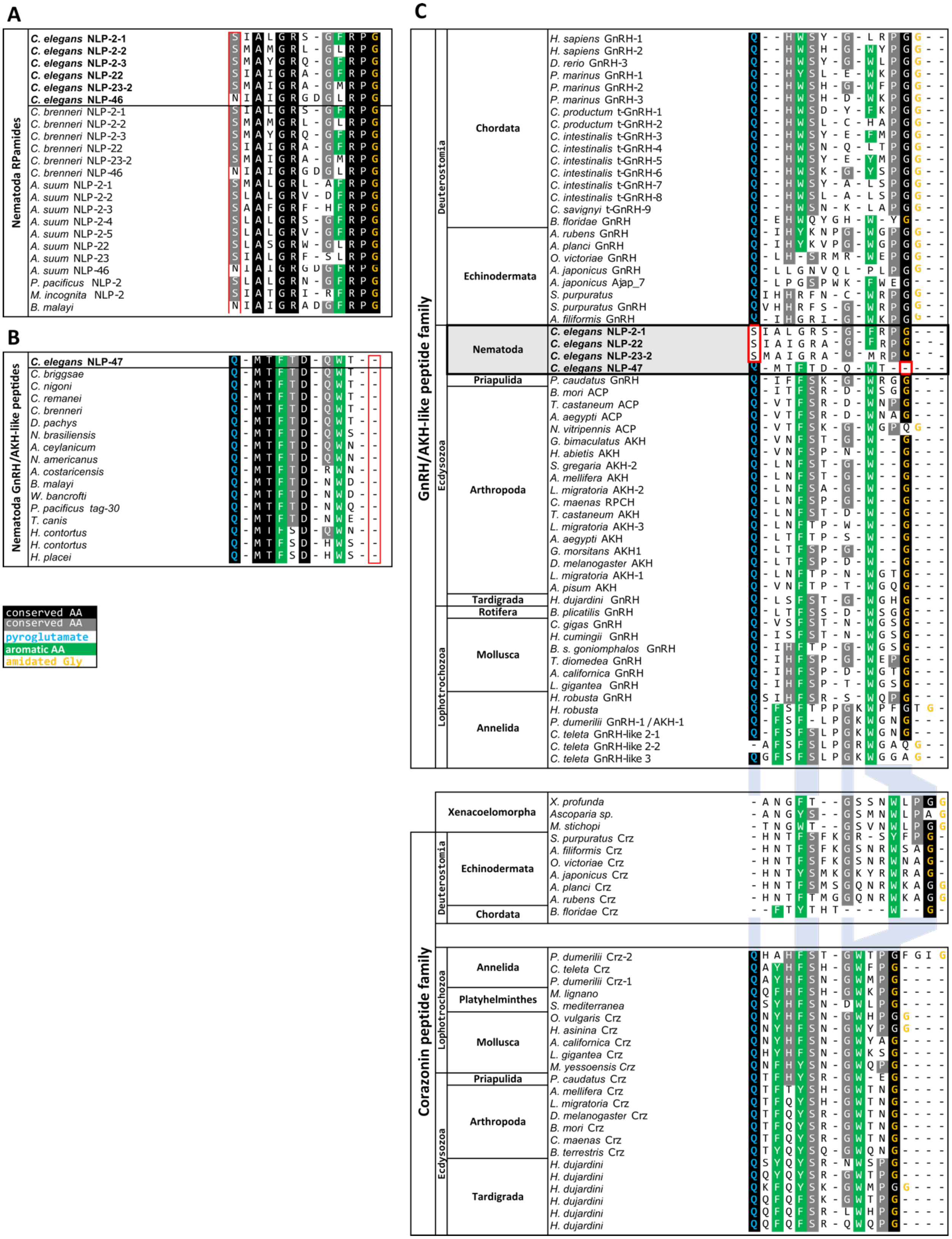
A
Nematoda RPamides
C. elegans NLP-2-1
C. elegans NLP-2-2
C. elegans NLP-2-3
C. elegans NLP-22
C. elegans NLP-23-2
C. elegans NLP-46
C. brenneri NLP-2-1
C. brenneri NLP-2-2
C. brenneri NLP-2-3
C. brenneri NLP-22
C. brenneri NLP-23-2
C. brenneri NLP-46
A. suum NLP-2-1
A. suum NLP-2-2
A. suum NLP-2-3
A. suum NLP-2-4
A. suum NLP-2-5
A. suum NLP-22
A. suum NLP-23
A. suum NLP-46
P. pacificus NLP-2
M. incognita NLP-2
B. malayi
B
Nematoda GnRH/AKH-like peptides
C. elegans NLP-47
C. briggsae
C. nigoni
C. remanei
C. brenneri
D. pachys
N. brasiliensis
A. ceylanicum
N. americanus
A. costaricensis
B. malayi
W. bancrofti
P. pacificus tag-30
T. canis
H. contortus
H. contortus
H. placei
conserved AA
conserved AA
pyroglutamate
aromatic AA
amidated Gly
C
GnRH/AKH-like peptide family
Deuterostomia
Chordata
H. sapiens GnRH-1
H. sapiens GnRH-2
D. rerio GnRH-3
P. marinus GnRH-1
P. marinus GnRH-2
P. marinus GnRH-3
C. productum t-GnRH-1
C. productum t-GnRH-2
C. intestinalis t-GnRH-3
C. intestinalis t-GnRH-4
C. intestinalis t-GnRH-5
C. intestinalis t-GnRH-6
C. intestinalis t-GnRH-7
C. intestinalis t-GnRH-8
C. savignyi t-GnRH-9
B. floridae GnRH
Echinodermata
A. rubens GnRH
A. planci GnRH
O. victoriae GnRH
A. japonicus GnRH
A. japonicus Ajap_7
S. purpuratus
S. purpuratus GnRH
A. filiformis GnRH
Nematoda
C. elegans NLP-2-1
C. elegans NLP-22
C. elegans NLP-23-2
C. elegans NLP-47
Ecdysozoa
Priapulida
P. caudatus GnRH
Arthropoda
B. mori ACP
T. castaneum ACP
A. aegypti ACP
N. vitripennis ACP
G. bimaculatus AKH
H. abietis AKH
S. gregaria AKH-2
A. mellifera AKH
L. migratoria AKH-2
C. maenas RPCH
T. castaneum AKH
L. migratoria AKH-3
A. aegypti AKH
G. morsitans AKH1
D. melanogaster AKH
L. migratoria AKH-1
A. pisum AKH
Tardigrada
H. dujardini GnRH
Lophotrochozoa
Rotifera
B. plicatilis GnRH
Mollusca
C. gigas GnRH
H. cumingii GnRH
B. s. goniomphalos GnRH
T. diomedea GnRH
A. californica GnRH
L. gigantea GnRH
Annelida
H. robusta GnRH
H. robusta
P. dumerilii GnRH-1 / AKH-1
C. teleta GnRH-like 2-1
C. teleta GnRH-like 2-2
C. teleta GnRH-like 3
Corazonin peptide family
Xenacoelomorpha
X. profunda
Ascoparia sp.
M. stichopi
Deuterostomia
Echinodermata
S. purpuratus Crz
A. filiformis Crz
O. victoriae Crz
A. japonicus Crz
A. planci Crz
A. rubens Crz
Chordata
B. floridae Crz
Lophotrochozoa
Annelida
P. dumerilii Crz-2
C. teleta Crz
P. dumerilii Crz-1
Platyhelminthes
M. lignano
S. mediterranea
Mollusca
O. vulgaris Crz
H. asinina Crz
A. californica Crz
L. gigantea Crz
M. yessoensis Crz
Ecdysozoa
Priapulida
P. caudatus Crz
Arthropoda
A. mellifera Crz
L. migratoria Crz
D. melanogaster Crz
B. mori Crz
C. maenas Crz
B. terrestris Crz
Tardigrada
H. dujardini
H. dujardini
H. dujardini
H. dujardini
H. dujardini
H. dujardini

Supplementary Fig. S5. Extensive alignment of RPamide and GnRH/AKH neuropeptide sequences

(A) Amino acid sequence alignment of nematode RPamides. Neuropeptide sequences were retrieved through a protein BLAST search restricted to nematodes using *C. elegans* NLP-2-1, NLP-2-2, NLP-2-3, NLP-22 and NLP-23-2 as a query. None of the RPamide peptide sequences contains an N-terminal pyroglutamate. **(B)** Amino acid sequence alignment of nematode GnRH/AKH peptides. Neuropeptide sequences were retrieved through a protein BLAST search restricted to nematodes using *C. elegans* NLP-47 as a query. All the known nematode GnRH/AKH-like sequences lack the C-terminal glycine amidation target. **(C)** Amino acid sequence alignment of diverse GnRH/AKH/Corazonin-like peptides across major animal phyla. Neuropeptide sequences were collected by BLASTp searches on the NCBI website and from literature [4–19]. For A-C, residues with a colored background are conserved in at least 40% of the sequences. Nearly identical residues (>80%) are depicted in black, conserved residues in grey and conserved aromatic residues in green. Pyroglutamate residues present at the N-termini are indicated in blue and glycine targets for amidation at the C-termini are indicated in orange. Absence of these terminal modifications in nematode RPamide or GnRH/AKH-like peptides, respectively, are indicated by red boxes. Hyphens indicate gaps. Faint blue shading between boxes in panel (C) indicates sequence similarity.

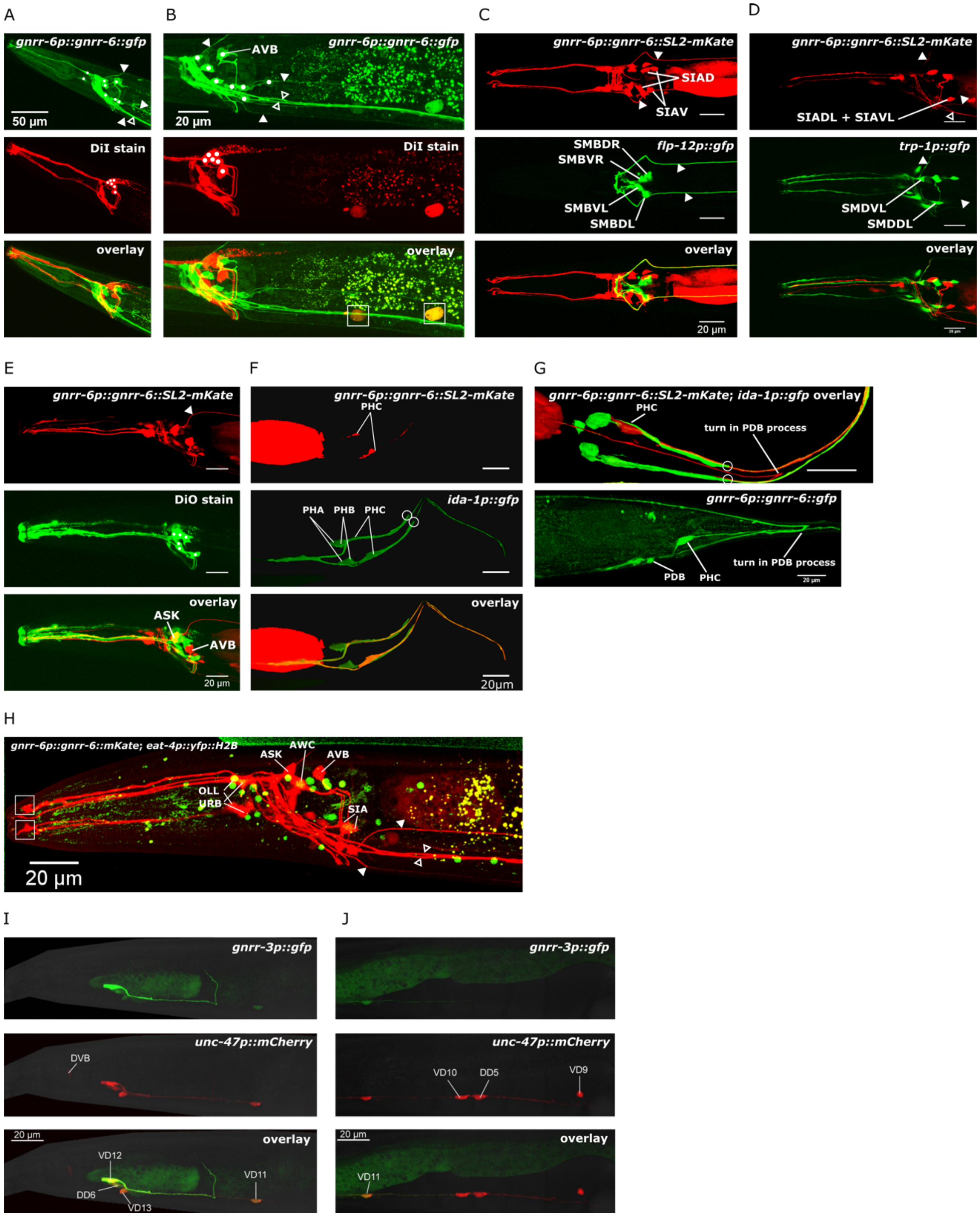

Supplementary Fig. S6. Expression patterns of *gnrr-3* and *gnrr-6*

(A-B) A translational reporter transgene for *gnrr-6* (*gnrr-6p::gnrr-6::gfp*) is expressed in sublateral motor neurons (indicated by filled white triangles) and AVB interneurons with processes in the ventral nerve cord (indicated by empty white triangles). Bottom panels show an overlay between red fluorescent DiI staining of specific amphid sensory neurons and GFP

signals for *gnrr-6*. No colocalization was observed for this dim green strain. The top and middle rows show separate channels for these reporters. White dots indicate cell nuclei. White boxes in the bottom panel indicate fluorescence from a coelomocyte co-injection marker. **(C-H)** A second translational reporter transgene for *gnrr-6* (*gnrr-6p::gnrr-6::sl2-mKate*) with brighter fluorescence confirms expression in sublateral motor neurons and AVB interneurons. Crossing with either a *[flp-12p::gfp]* **(C)** or a *[trp-1p::gfp]* marker strain **(D)** excluded respectively SMB and SMD neurons from expressing *gnrr-6*. Based on morphology and position, we identified SIA neurons as *gnrr-6* expressing cells. **(E-F)** We also confirmed *gnrr-6* expression in ASK and PHC sensory neurons based on colocalization with DiO staining **(E)** and crossing with a *[ida-1p::gfp]* marker strain **(F)**, respectively. **(G)** Both *gnrr-6p::gnrr-6::SL2::mKate* (upper panel) *and gnrr-6p::gnrr-6::gfp* (lower panel) reporter strains show the characteristic process of the PDB neuron, which turns dorsally posterior to the phasmid openings (indicated by white circles in panels F and G). **(H)** The morphology and relative position of fluorescent cells in the *gnrr-6* reporter strain compared to the expression of the *[eat-4p::yfp::H2B]* nuclear-localized glutamatergic reporter transgene[21] suggests that also OLL, URB and AWC sensory neurons express *gnrr-6.* White squares indicate the characteristic wing-shaped dendritic ending of AWC neurons. **(I-J)** A transcriptional reporter transgene for *gnrr-3* (*gnrr-3p::gfp*) reveals expression in several inhibitory GABAergic motor neurons of the ventral nerve cord (VNC) in the tail and posterior body. To validate cell identifications, animals expressing the *gnrr-3* reporter transgene were crossed with a red GABAergic reporter strain (*unc-47p::mcherry*). Bottom panels show an overlay between GFP signals for *gnrr-3* and red fluorescence for the GABAergic reporter. The top and middle panels show the separate channels for the GABAergic reporter and the *gnrr-3* reporter transgene, respectively. Expression for *gnrr-3* is seen in VD12, VD13, DD6 and VD11.

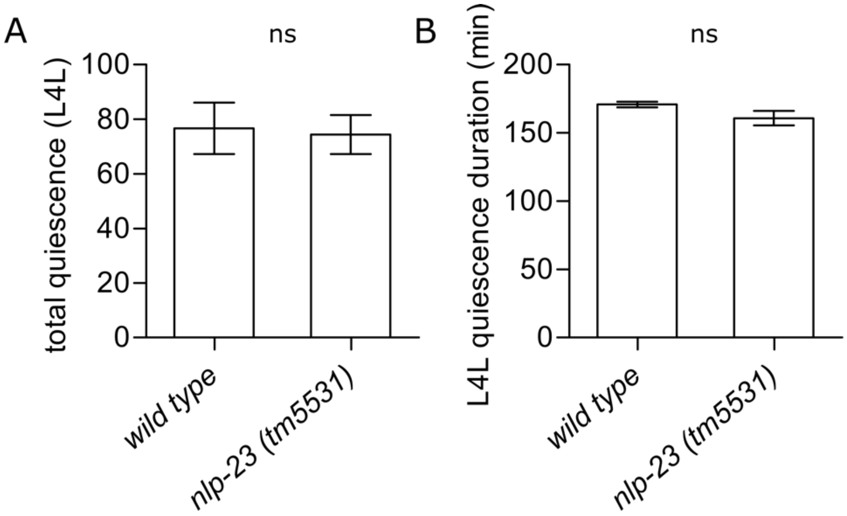

Supplementary Fig. S7. *nlp-23* mutants display normal quiescence during lethargus

(A) Average total quiescence during L4 lethargus (L4L) and **(B)** average quiescence duration of L4L of *nlp-23* mutants (N = 6 animals) are not significantly different from wild type animals. Error bars around mean indicate SEM. Paired student's two-tailed t-test; ns, not significant ($P > 0.05$).

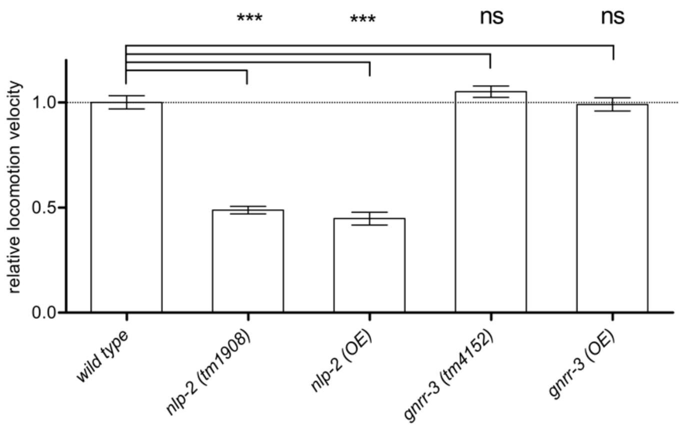

Supplementary Fig. S8. *nlp-2* signaling affects locomotion speed in adults

Average forward locomotion speed of adult animals for mutants of *nlp-2* and *gnrr-3*, and animals overexpressing *nlp-2* or *gnrr-3*. Speed is plotted relative to the forward locomotion speed of wild-type animals (dotted line). One-way ANOVA and Tukey post-hoc comparison; ***$P < 0.001$; ns, not significant ($P < 0.05$); N > 100 animals; error bars indicate SEM.

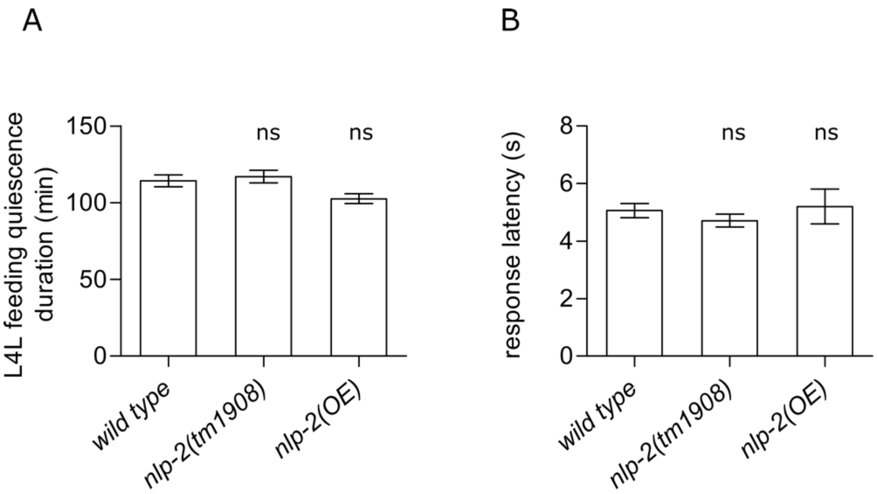

Fig. S9. ***nlp-2* does not modulate the duration of feeding quiescence or blue light responsiveness during L4 lethargus**

(A) Average feeding quiescence duration and **(B)** response latency to blue light during L4 lethargus (L4L) of *nlp-2* mutants, and animals overexpressing *nlp-2*. One-way ANOVA and Tukey post-hoc comparison; ns, not significant ($P > 0.05$); N > 20 animals; error bars represent SEM.

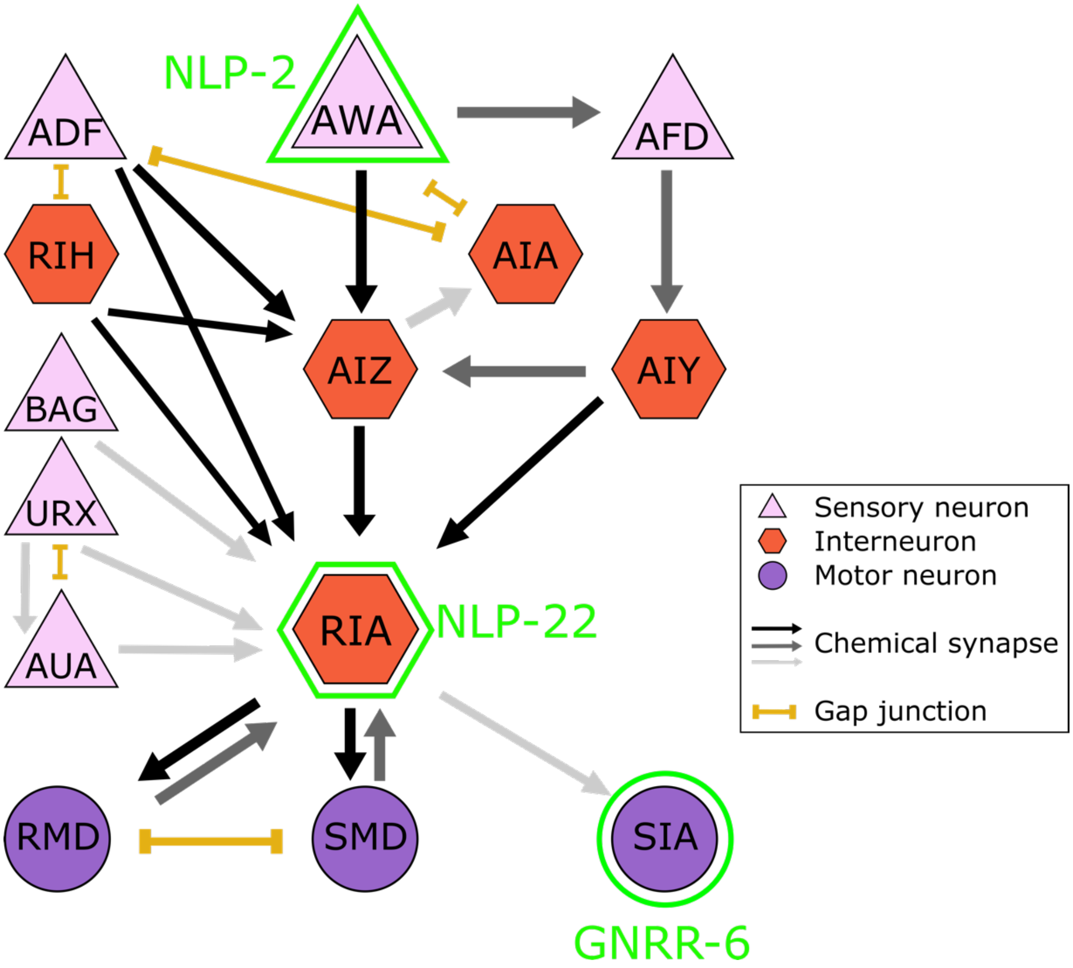

Supplementary Fig. S10 Network diagram of the major connections with RPamide expressing neurons controlling sleep-wake states

The wake-promoting NLP-2 neuropeptides are expressed in the AWA olfactory neurons which primarily project to AIA and AIZ interneurons[22]. The AIZ neurons are known to decrease forward locomotion bouts, which is opposed by AIY signaling [23,24]. Downstream of AIZ and AIY, the RIA interneurons integrate odor/pheromone (AWA), thermal (AFD) and oxygen (BAG, URX, AUA, ADF) stimuli collectively to control locomotion behavior appropriately [25–28]. Somnogenic NLP-22 neuropeptides are released by these RIA interneurons, which are pre-synaptic to sublateral motor neurons (SMD, SIA) involved in control of locomotion and quiescence behavior [29–32]. The SIA sublateral motor neurons express *gnrr-6* and are known to have a function in turning behavior during sleep[30].

Supplementary Table S1. Transgenic strains used in this study

Strain	Genotype	Description
NQ251	*qnIs142 [hsp16.2p::nlp-22; hsp16.2p::gfp; myo-2p::mCherry; unc-119(+)]*	Heat-shock inducible *nlp-22* expression
LSC559	*gnrr-3 (tm4152); qnIs142 [hsp16.2p:nlp-22; hsp16.2p::gfp; myo-2p::mCherry; unc-119(+)]*	Heat-shock inducible *nlp-22* expression in *gnrr-3 (-)* background
LSC1520	*gnrr-6 (ok3465); qnIs142 [hsp16.2p:nlp-22; hsp16.2p::gfp; myo-2p::mCherry; unc-119(+)]*	Heat-shock inducible *nlp-22* expression in *gnrr-6 (-)* background
NQ644	*qnEx343 [gnrr-3p::gnrr-3; myo-2p::mCherry]*	*gnrr-3 (OE)*
LSC1254	*qnEx363 [nlp-2p::nlp-2; myo-2p::mCherry]*	*nlp-2 (OE)*
NQ776	*gnrr-3 (tm4152); qnEx363 [nlp-2p::nlp-2; myo-2p::mCherry]*	*nlp-2 (OE); gnrr-3 (-)*
LSC1903	*gnrr-6 (ok3465); qnEx363 [nlp-2p::nlp-2; myo-2p::mCherry]*	*nlp-2 (OE); gnrr-6 (-)*
NQ694	*qnEx365 [nlp-23p::nlp-23; myo-2p::mCherry]*	*nlp-23 (OE)*
NQ774	*qnEx423 [nlp-2p::gfp; glr-3p::mCherry; rol-6(d)]*	*nlp-2* reporter strain
LSC1298	*lstEx682[odr-10p::mCherry::3'UTR odr-10; unc-122p::gfp]*	red AWA marker strain[33]
LSC1091	*lstEx556[gnrr-3p::gnrr-3::gfp; unc-122p::mCherry]*	*gnrr-3* reporter strain
LSC1258	*lstEx556 [gnrr-3p::gnrr-3::gfp; unc-122p::mCherry]; wpIs36 [unc-47p::mCherry]*	*gnrr-3* reporter strain and red GABA marker strain[34]
LSC1687	*lstEx1023[gnrr-6p::gnrr-6::gfp; unc-122p::mCherry]*	*gnrr-6* reporter strain
LSC1904	*lstEx1048[gnrr-6p::gnrr-6::sl2-mKate; unc-122p::gfp]*	*gnrr-6* reporter strain
AQ3642	*ynIs25[flp-12p::gfp; rol-6d]*	SMB marker strain[35]
AQ3848	*kyIs123[trp-1p::gfp]*	SMD marker strain[31]
BL5717	*inIs179[ida-1p::gfp] II; him-8(e1489) IV.*	PHC marker strain[36,37]
OH12312	*otIs388[eat-4(fosmid)::sl2::yfp::H2B; pha-1(e2123)]; him-5(e1490)*	glutamatergic marker strain[21]
AQ2529	*ljEx286[sra-11p::YC3.60]*	AVB marker strain[38]

Supplementary Table S2. Genbank accession numbers of protein dataset used for phylogenetic analysis of GnRH/AKH receptor sequences

deuterostomian GnRH receptors	NP_001028994.1, NP_001028997.1, NP_001028996.1, AAW70562.1, XP_002606331.1, XP_002606330.1, NP_000397.1, XP_003965821.1, XP_003977749.1, XP_003967097.1, XP_003969466.1, XP_003969621.1, NP_001116990.1, NP_001116991, NP_001116992.1, AAQ04564.1, ADL14592.1, ABO77118.1, XP_005174743.1, ABQ08716.1, NP_001138451.1, and NP_001091663.1
bilaterian corazonin receptors	XP_006820827, XP_009044166.1, ELT93721.1, XP_002606367.1, ACC68668.1, NP_001137393.1, NP_648571.1, NP_001127719.1, and XP_321555.1
protostomian GnRH/AKH receptors	ACD75498.1, XP_003245942.1, ELU01220.1, ELT92261.1, EOG63VBPF, ELT96363.1, ELU00909.1, NP_477387.1, NP_001076809.1, NP_001035354.1, NP_001037049.1, ABD60146.1, NP_001127745.1, NP_001280549.1, ABX52399.1, and NP_001164571.1
nematode GnRH/AKH-like receptors	NP_001249720.1, NP_001256583.1, NP_509685.2, NP_001024452.1, NP_504228.2, NP_509865.1, NP_509866.2, NP_502887.1, XP_001898606.1, XP_001898983.1, XP_001897938.1, XP_002639368.1, XP_002637834.1, XP_002643743.1, XP_002644741.1, XP_002647239.1, XP_002644017.1, XP_002644016.1, and CAP32453.2
AVP and NPS receptors	XP_002595923.1, XP_002741531.1, ELU01967.1, AAH30197.1, XP_002600070.1, XP_006815767.1, ELU14444.1, NP_996297.3, ELU12393.1, NP_997055.1, ENSPMAT00000003799, XP_002605430.1, XP_006813812.1, NP_493193.1, NP_510477.1, XP_002586963.1, NP_000045.1, ENSPMAT00000008459, NP_000698.1, CAA46097.1, XP_002735546.2, ELU02228.1, and EFX69326.1

Supplementary Table S3. Genbank accession numbers of peptide alignment in Fig. 3

Clade	Superphylum	Phylum	Species	Accession number(s) or reference
Protostomia	Ecdysozoa	Nematoda	***Caenorhabditis elegans***	NP_508426 NP_508424 NP_508425 NP_001024176 NP_500770
Protostomia	Ecdysozoa	Nematoda	***Caenorhabditis brenneri***	EGT30199 EGT30310 EGT30508 EGT55156 CAP37115
Protostomia	Ecdysozoa	Nematoda	***Caenorhabditis briggsae***	CAP37115
Protostomia	Ecdysozoa	Nematoda	***Caenorhabditis nigoni***	PIC33286
Protostomia	Ecdysozoa	Nematoda	***Caenorhabditis remanei***	EGT40149
Protostomia	Ecdysozoa	Nematoda	***Ascaris suum***	AWJ58397.1 AIB09454.1 AWJ58398.1 AWJ58399.1
Protostomia	Ecdysozoa	Nematoda	***Pristionchus pacificus***	PDM64822 PDM72575
Protostomia	Ecdysozoa	Nematoda	***Meloidogyne incognita***	APU93371
Protostomia	Ecdysozoa	Nematoda	***Brugia malayi***	XP_001901900 VDM21668
Protostomia	Ecdysozoa	Nematoda	***Diploscapter pachys***	PAV78745
Protostomia	Ecdysozoa	Nematoda	***Nippostrongylus brasiliensis***	VDL73364
Protostomia	Ecdysozoa	Nematoda	***Ancylostoma ceylanicum***	EYC31271
Protostomia	Ecdysozoa	Nematoda	***Necator americanus***	XP_013290647
Protostomia	Ecdysozoa	Nematoda	***Angiostrongylus costaricensis***	VDM58181
Protostomia	Ecdysozoa	Nematoda	***Wuchereria bancrofti***	VDM21668
Protostomia	Ecdysozoa	Nematoda	***Toxocara canis***	KHN74123
Protostomia	Ecdysozoa	Nematoda	***Haemonchus contortus***	CDJ98198 CDJ98199
Protostomia	Ecdysozoa	Nematoda	***Haemonchus placei***	VDO27384
Protostomia	Ecdysozoa	Priapulida	***Priapulus caudatus***	Reference Hauser & Grimmelikhuijzen, 2014
Protostomia	Ecdysozoa	Arthropoda	***Apis mellifera***	XP_006563609 NP_001012981
Protostomia	Ecdysozoa	Arthropoda	***Locusta migratoria***	P55319.1 P08379 P19872 AKN21243
Protostomia	Ecdysozoa	Arthropoda	***Drosophila melanogaster***	NP_523918.1 NP_524350
Protostomia	Ecdysozoa	Arthropoda	***Bombyx mori***	NP_001127713 XP_021204062
Protostomia	Ecdysozoa	Arthropoda	***Carcinus maenas***	AAB28133.1 AVA26882
Protostomia	Ecdysozoa	Arthropoda	***Bombus terrestris***	Reference Roch *et al.* , 2011
Protostomia	Ecdysozoa	Arthropoda	***Tribolium castaneum***	EFA12888 NP_001107797
Protostomia	Ecdysozoa	Arthropoda	***Aedes aegypti***	EAT37004 XP_001655817
Protostomia	Ecdysozoa	Arthropoda	***Nasonia vitripennis***	NP_001161199
Protostomia	Ecdysozoa	Arthropoda	***Gryllus bimaculatus***	P67785
Protostomia	Ecdysozoa	Arthropoda	***Hylobius abietis***	Reference Marchal *et al* ., 2018
Protostomia	Ecdysozoa	Arthropoda	***Schistocerca gregaria***	AAB20138
Protostomia	Ecdysozoa	Arthropoda	***Glossina morsitans***	AEH25941
Protostomia	Ecdysozoa	Arthropoda	***Acyrthosiphon pisum***	NP_001243520
Protostomia	Ecdysozoa	Tardigrada	***Hypsibius dujardini***	OQV25418 OWA52622.1 OQV24539.1 OQV20368.1 OQV15187 OWA51386 OWA52075
Protostomia	Lophotrochozoa	Rotifera	***Brachionus plicatilis***	RNA39930
Protostomia	Lophotrochozoa	Annelida	***Helobdella robusta***	XP_009015960 Reference Li *et al.* , 2016
Protostomia	Lophotrochozoa	Annelida	***Capitella teleta***	ELU16520 ELU06546 Reference Li *et al.* , 2016
Protostomia	Lophotrochozoa	Annelida	***Platynereis dumerilii***	AHB62361 Reference Williams *et al.* , 2017
Protostomia	Lophotrochozoa	Platyhelminthes	***Macrostomum lignano***	PAA82237
Protostomia	Lophotrochozoa	Platyhelminthes	***Schmidtea. mediterranea***	ADC84439
Protostomia	Lophotrochozoa	Mollusca	***Crassostrea gigas***	AKA95279
Protostomia	Lophotrochozoa	Mollusca	***Hyriopsis cumingii***	Reference Zandawala *et al.* , 2018
Protostomia	Lophotrochozoa	Mollusca	***Bithynia siamensis goniomphalos***	Reference Zandawala *et al.* , 2018
Protostomia	Lophotrochozoa	Mollusca	***Tritonia diomedea***	Reference Hauser & Grimmelikhuijzen, 2014
Protostomia	Lophotrochozoa	Mollusca	***Octopus vulgaris***	BAB86782
Protostomia	Lophotrochozoa	Mollusca	***Haliotis asinina***	AKR13998
Protostomia	Lophotrochozoa	Mollusca	***Aplysia californica***	NP_001268793 NP_001191482.1
Protostomia	Lophotrochozoa	Mollusca	***Lottia gigantea***	Reference Hauser & Grimmelikhuijzen, 2014 Reference Roch *et al.* , 2011
Protostomia	Lophotrochozoa	Mollusca	***Mizuhopecten yessoensis***	XP_021347551
Deuterostomia		Chordata	***Homo sapiens***	NP_000816 NP_001492
Deuterostomia		Chordata	***Danio rerio***	NP_878307
Deuterostomia		Chordata	***Petromyzon marinus***	AF144480_1 ABE66462 P30948
Deuterostomia		Chordata	***Ciona productum***	Reference Tello *et al.* , 2005
Deuterostomia		Chordata	***Ciona intestinalis***	NP_001027799 AAP06795
Deuterostomia		Chordata	***Ciona savignyi***	Reference Tello *et al.* , 2005
Deuterostomia		Chordata	***Branchiostoma floridae***	XP_002603207 AHE40598 Reference Zandawala *et al.* , 2018
Deuterostomia		Echinodermata	***Strongylocentrotus purpuratus***	XP_011682962 XP_800179 Reference Semmens *et al.* , 2017
Deuterostomia		Echinodermata	***Amphiura filiformis***	Reference Zandawala, 2017
Deuterostomia		Echinodermata	***Ophionotus victoriae***	ASK86273 ASK86243
Deuterostomia		Echinodermata	***Apostichopus japonicus***	AWU78767 AWU78768 AWU78766
Deuterostomia		Echinodermata	***Acanthaster planci***	Reference Smith *et al.* ,2017
Deuterostomia		Echinodermata	***Asterias rubens***	ALJ99954 ALJ99955
		Xenacoelomorpha	***Xenoturbella profunda***	Reference Thiel *et al.* , 2018
		Xenacoelomorpha	***Ascoparia sp.***	Reference Thiel *et al.* , 2018
		Xenacoelomorpha	***Meara stichopi***	Reference Thiel *et al.* , 2018

Supplementary Table S4. Primer sequences for overexpression constructs

Primer	Sequence (5'-3')
gnrr-3 forward	AGGATAAGTGTCACTCTTCGGAC
gnrr-3 reverse	TCATTTCCTAAACAACCCAGAC
nlp-2 forward	ATGACACGTACTATATTGTTCAAAGATG
nlp-2 reverse	ATATAGAATTTATTCAATTGTATGGAGA

Supplementary Table S5. Primer sequences for fluorescent reporter constructs

Primer	Sequence (5'-3')
nlp-2p A	ATGACACGTACTATATTGTTCAAAGATG
nlp-2p A'	GACAACGTGATTTTGAACAAAAAC
nlp-2p::gfp B	AAAAGTTCTTCTCCTTTACTCATTGCTCGCATTTCTCGCGTTGT
nlp-2p::gfp C	TCCCAACAACGCGAGAAATGCGAGCAATGAGTAAAGGAGAAGAACTTTT
gfp D'	GAGAAGTTTTTTGATAATAACAAAAATAGG
gfp D	AAAAGAAGCTAAAAAACAAAGAAATTA
gnrr-3p A	AGGATAAGTGTCACTCTTCGGAC
gnrr-3p A'	GATTAGATGATTCGCTTATCTCCGAAG
gnrr-3p::gfp B	TCCTGAAAATGTTCTATGTTATGTTTCTGAAAAGTTTCACAATTG
gnrr-3p::gfp C	CAATTGTGAAACTTTTCAGAAACATAACATAGAACATTTTCAGGA
gnrr-6p::gnrr-6::gfp Fw	CGAACCTCTTTCGGCAGCGGATTG
gnrr-6p::gnrr-6::gfp Rv	ACCTCGACGGTTTCTTGCTACTTG
gnrr-6p Fw Gateway	GGGGACAACTTTGTATAGAAAAGTTGCGAACCTCTTTCGGCAGCG
gnrr-6p Rv Gateway	GGGGACTGCTTTTTTGTACAAACTTGTGTAACTTGAACAAACGAAGTGTTG
gnrr-6 Fw Gateway	GGGGACAAGTTTGTACAAAAAAGCAGGCTtttcagaaaaATGTTTGACAGGCAGATGGAG
gnrr-6 Rv Gateway	GGGGACCACTTTGTACAAGAAAGCTGGGTCTAGTTTCCAGTTGAGAAACCG

References from Supplementary Figures

1. Krogh, A., Larsson, È., Heijne, G. Von & Sonnhammer, E. L. L. Predicting Transmembrane Protein Topology with a Hidden Markov Model : Application to Complete Genomes. *J. Mol. Biol.* **305**, 567–580 (2001).
2. Edgar, R. C. MUSCLE : multiple sequence alignment with high accuracy and high throughput. *Nucleic Acids Res.* **32**, 1792–1797 (2004).
3. Kumar, S., Stecher, G. & Tamura, K. MEGA7 : Molecular Evolutionary Genetics Analysis Version 7 . 0 for Bigger Datasets. *Mol. Biol. Evol.* **33**, 1870–1874 (2016).
4. Conzelmann, M. *et al.* The neuropeptide complement of the marine annelid *Platynereis dumerilii*. *BMC Genomics* **14**, 906 (2013).
5. Roch, G. J., Busby, E. R. & Sherwood, N. M. Evolution of GnRH: Diving deeper. *Gen. Comp. Endocrinol.* **171**, 1–16 (2011).
6. Li, S. *et al.* Adipokinetic hormones and their G protein-coupled receptors emerged in Lophotrochozoa. *Sci. Rep.* **6**, 32789 (2016).
7. Hauser, F. & Grimmelikhuijzen, C. J. P. Evolution of the AKH/corazonin/ACP/GnRH receptor superfamily and their ligands in the Protostomia. *Gen. Comp. Endocrinol.* **209**, 35–49 (2014).
8. Alexander, J. L. *et al.* Functional characterization and signaling systems of corazonin and red pigment concentrating hormone in the green shore crab, *Carcinus maenas*. *Front. Neurosci.* **11**, 752 (2018).
9. Smith, M. K. *et al.* The neuropeptidome of the Crown-of-Thorns Starfish, *Acanthaster planci*. *J. Proteomics* **165**, 61–68 (2017).
10. Hayakawa, E. *et al.* A combined strategy of neuropeptide predictions and tandem mass spectrometry identifies evolutionarily conserved ancient neuropeptides in the sea anemone *Nematostella vectensis*. *PLoS One* **14**, e0215185 (2019).
11. Chen, M., Talarovicova, A., Zheng, Y., Storey, K. B. & Elphick, M. R. Neuropeptide precursors and neuropeptides in the sea cucumber *Apostichopus japonicus*: a genomic, transcriptomic and proteomic analysis. *Sci. Rep.* **9**, 8829 (2019).
12. Semmens, D. C. & Elphick, M. R. The evolution of neuropeptide signalling: Insights from echinoderms. *Brief. Funct. Genomics* **16**, 288–298 (2017).
13. Koziol, U. Precursors of neuropeptides and peptide hormones in the genomes of tardigrades. *Gen. Comp. Endocrinol.* **267**, 116–127 (2018).
14. Marchal, E. *et al.* Analysis of Peptide Ligand Specificity of Different Insect Adipokinetic Hormone Receptors. *Int. J. Mol. Sci.* **19**, 542 (2018).
15. Williams, E. A. *et al.* Synaptic and peptidergic connectome of a neurosecretory center in the annelid brain. *Elife* **6**, e26349 (2017).
16. Zandawala, M. *et al.* Discovery of novel representatives of bilaterian neuropeptide families and reconstruction of neuropeptide precursor evolution in ophiuroid echinoderms. *Open Biol.* **7**, 170129 (2017).
17. Zandawala, M., Tian, S. & Elphick, M. R. The evolution and nomenclature of GnRH-type and corazonin-type neuropeptide signaling systems. *Gen. Comp. Endocrinol.* **264**, 64–77 (2018).
18. Thiel, D., Franz-Wachtel, M., Aguilera, F. & Hejnol, A. Xenacoelomorph neuropeptidomes reveal a major expansion of neuropeptide systems during early bilaterian evolution. *Mol. Biol. Evol.* **35**, 2528–2543 (2018).
19. Roch, G. J., Tello, J. A. & Sherwood, N. M. At the transition from invertebrates to vertebrates, a novel gnrh-like peptide emerges in amphioxus. *Mol. Biol. Evol.* **31**, 765–778 (2014).
20. Taylor, S. R. *et al.* Expression profiling of the mature *C. elegans* nervous system by single-cell RNA-Sequencing. *bioRxiv* 737577 (2019). doi:10.1101/737577
21. Serrano-Saiz, E. *et al.* Modular control of glutamatergic neuronal identity in *C. elegans* by distinct homeodomain proteins. *Cell* **155**, 659 (2013).
22. Larsch, J. *et al.* A Circuit for Gradient Climbing in *C. elegans* Chemotaxis. *Cell Rep.* **12**, 1748–1760 (2015).
23. Tsalik, E. L. & Hobert, O. Functional mapping of neurons that control locomotory behavior in *Caenorhabditis elegans*. *J. Neurobiol.* **56**, 178–197 (2003).
24. de Bono, M. & Villu Maricq, A. Neuronal substrates od complex behaviors in *C. elegans*. *Annu. Rev. Neurosci.* **28**, 451–501 (2005).
25. Mori, I. & Ohshima, Y. Neural regulation of thermotaxis in *C. elegans*. *Nature* **376**, 344–348 (1995).
26. Ha, H. ick *et al.* Functional Organization of a Neural Network for Aversive Olfactory Learning in *Caenorhabditis elegans*. *Neuron* **68**, 1173–1186 (2010).
27. Guillermin, M. L., Carrillo, M. A. & Hallem, E. A. A Single Set of Interneurons Drives Opposite Behaviors in *C. elegans*. *Curr. Biol.* **27**, 2630-2639.e6 (2017).
28. Hendricks, M. & Zhang, Y. Complex RIA calcium dynamics and its function in navigational behavior. *Worm* **2**, e25546 (2013).
29. Nelson, M. D. *et al.* The neuropeptide NLP-22 regulates a sleep-like state in *Caenorhabditis elegans*. *Nat. Commun.* **4**, 2846 (2013).
30. Schwarz, J. & Bringmann, H. Analysis of the NK2 homeobox gene ceh-24 reveals sublateral motor neuron control of left-right turning during sleep. *Elife* **6**, 1–25 (2017).
31. Yeon, J. *et al.* A sensory-motor neuron type mediates proprioceptive coordination of steering in *C. elegans* via two TRPC channels. *PLOS Biol.* **16**, e2004929 (2018).
32. Ouellette, M.-H., Desrochers, M. J., Gheta, I., Ramos, R. & Hendricks, M. A Gate-and-Switch Model for Head Orientation Behaviors in *Caenorhabditis elegans*. *Eneuro* **5**, ENEURO.0121-18.2018 (2018).
33. Sengupta, P., Chou, J. H. & Bargmann, C. I. odr-10 Encodes a seven transmembrane domain olfactory receptor required for responses to the odorant diacetyl. *Cell* **84**, 899–909 (1996).
34. Firnhaber, C. & Hammarlund, M. Neuron-Specific Feeding RNAi in C. elegans and Its Use in a Screen for Essential Genes Required for GABA Neuron Function. *PLoS Genet.* **9**, e1003921 (2013).
35. Kim, J. *et al.* The Evolutionarily Conserved LIM Homeodomain Protein LIM-4/LHX6 Specifies the Terminal Identity of a Cholinergic and Peptidergic C. elegans Sensory/Inter/Motor Neuron-Type. *PLoS Genet.* **11**, 1–27 (2015).
36. Estevez, A. O. *et al.* Selenium induces cholinergic motor neuron degeneration in Caenorhabditis elegans. *Neurotoxicology* **33**, 1021–1032 (2012).
37. Zahn, T. R., Macmorris, M. A., Dong, W., Day, R. & Hutton, J. C. IDA-1, a Caenorhabditis elegans homolog of the diabetic autoantigens IA-2 and phogrin, is expressed in peptidergic neurons in the worm. *J. Comp. Neurol.* **429**, 127–143 (2001).
38. Troemel, E. R., Chou, J. H., Dwyer, N. D., Colbert, H. A. & Bargmann, C. I. Divergent seven transmembrane receptors are candidate chemosensory receptors in C. elegans. *Cell* **83**, 207–218 (1995).
